# Heart Failure Medication Withdrawal in Patients With Improved Cardiac Function After Atrial Fibrillation Ablation: The DEFINITION-AF Pilot Randomized Clinical Trial

**DOI:** 10.1001/jamanetworkopen.2026.20145

**Published:** 2026-06-26

**Authors:** Sitong Li, Yidan Sun, Yiwei Lai, Hui Wang, Jiancheng Han, Yuge Zhang, Mingyang Gao, Jue Wang, Jingrui Zhang, Liu He, Jing Cui, Xueyuan Guo, Song Zuo, Xiaoxia Liu, Nian Liu, Songnan Li, Ning Zhou, Ribo Tang, Deyong Long, Caihua Sang, Xin Du, Jianzeng Dong, Changsheng Ma

**Affiliations:** 1 Department of Cardiology, Beijing Anzhen Hospital, Capital Medical University, Beijing, China; 2 Department of Radiology, Beijing Anzhen Hospital, Capital Medical University, Beijing, China; 3 Maternal-Fetal Medicine Center in Fetal Heart Disease, Beijing Anzhen Hospital, Capital Medical University, Beijing, China

## Abstract

**Importance:**

Withdrawal of guideline-directed medical therapy (GDMT) for heart failure (HF) is common after atrial fibrillation (AF) catheter ablation and recovery of cardiac function, but safety remains uncertain.

**Objective:**

To assess the feasibility and safety of phased GDMT withdrawal in patients with AF with highly suspected AF-mediated cardiomyopathy after catheter ablation.

**Design, Setting, and Participants:**

This open-label pilot randomized clinical trial included adult patients who were enrolled and randomized from April 13, 2023, to September 19, 2024, at Beijing Anzhen Hospital, China, with a 6-month follow-up. Eligible patients were those with suspected AF-mediated cardiomyopathy at 3 months after ablation, defined by sinus rhythm at 3 months after ablation, absence of other suspected cardiomyopathies, improvement of left ventricular ejection fraction (LVEF) from 45% or less to 55% or more, normalized LV end-diastolic diameter (LVEDD), N-terminal pro–brain natriuretic peptide (NT-proBNP) levels less than 250 ng/L, and no HF symptoms or signs.

**Interventions:**

Patients were randomly assigned in a 1:1 ratio to phased GDMT withdrawal or GDMT continuation.

**Main Outcomes and Measures:**

The primary end point was HF deterioration, defined as LVEF decline more than 10% to less than 55%, LVEDD increase more than 10% and beyond normal, NT-proBNP levels doubling to more than 400 ng/L, or worsening HF signs or symptoms. Secondary outcomes included cardiovascular events, changes in echocardiographic and cardiac magnetic resonance (CMR) parameters, NT-proBNP levels, Kansas City Cardiomyopathy Questionnaire-12 (KCCQ-12) scores, atrial arrhythmia recurrence, and adverse drug events.

**Results:**

Among 50 patients enrolled and randomized, 47 completed follow-up (median [IQR] age, 56.0 [48.0-60.5] years; 37 males [78.7%]), including 23 (48.9%) in the GDMT withdrawal group and 24 (51.1%) in the continuation group. HF deterioration occurred in 3 patients with GDMT withdrawal (13.0%) and 0 with GDMT continuation (0%) (*P* = .11). Early GDMT reinitiation in 3 patients with HF deterioration showed recovery of LVEF or NT-proBNP. No cardiovascular events occurred. Median (IQR) echocardiographic (LVEF: 0% [−3.0% to 3.5%] vs 1.5% [−5.0% to 5.0%]), CMR (LVEF: −2.1% [−7.2% to 4.6%] vs 2.9% [−2.6% to 7.4%]), and KCCQ-12 score (0 [0 to 0.5] vs 0) changes were similar between groups. Median (IQR) NT-proBNP levels declined more in the GDMT continuation group than the withdrawal group (−25.7 [−33.6 to −6.7] pg/mL vs 2.7 [−21.4 to 24.2] pg/mL; *P* = .03). Adverse drug events were more frequent in the GDMT continuation group than the withdrawal group (5 [20.8%] vs 0 [0%]; *P* = .050). Arrhythmia recurrence rates were comparable (GDMT withdrawal: 3 [13.0%] vs continuation: 3 [12.5%]).

**Conclusions and Relevance:**

In this pilot randomized clinical trial of carefully selected patients with AF with normalized cardiac function and sinus rhythm after catheter ablation, 13% of patients with GDMT withdrawal experienced HF deterioration, whereas drug-related complications were more common in the continuation group, suggesting that further studies are needed to determine whether GDMT can be safely discontinued in this population.

**Trial Registration:**

Chinese Clinical Trial Registry Identifier: ChiCTR2300077439

## Key Points

**Question:**

Is discontinuation of guideline-directed medical therapy (GDMT) after catheter ablation feasible and safe in patients with atrial fibrillation (AF) with improved cardiac function?

**Findings:**

In this randomized clinical trial of 50 patients with AF and heart failure (HF) with improved ejection fraction (suspected with AF-mediated cardiomyopathy), phased GDMT withdrawal in those with normalized left ventricular ejection fraction and sinus rhythm resulted in HF deterioration in 3 of 23 patients (13%) compared with none in the continuation group. This was not a statistically significant difference.

**Meaning:**

Although this study did not find significantly more HF deterioration in patients who discontinued GDMT vs those who continued, further studies are needed to determine whether GDMT can be safely discontinued in this population.

## Teaser

This pilot randomized clinical trial assesses whether phased withdrawal of heart failure medication in patients who have undergone catheter ablation for atrial fibrillation (AF) and experienced improvements in cardiac function is feasible and safe.

## Introduction

Atrial fibrillation (AF) and heart failure (HF) commonly coexist, with each condition exacerbating the other and worsening outcomes. AF contributes to HF progression through mechanisms such as tachycardia, irregular ventricular rhythm, atrioventricular dyssynchrony, and impaired atrial systole.^1^ In AF-mediated cardiomyopathy (AMC), characterized by a reversible etiology of HF, cardiac function often improves markedly after restoration of sinus rhythm.^2^ Catheter ablation has been shown to improve left ventricular ejection fraction (LVEF) more effectively than rate control in patients with AF and nonischemic cardiomyopathy^3^ and is recommended as first-line therapy for patients likely to have AMC.^4,5,6^ However, the optimal long-term management of guideline-directed medical therapy (GDMT) of HF in these patients remains unclear.^7^

Prolonged GDMT may cause adverse effects and increase costs. In clinical practice, HF medication discontinuation is common in patients with AF with improved cardiac function following ablation,^8^ yet its safety has not been well evaluated. Therefore, we conducted a pilot randomized clinical trial to assess the feasibility and safety of phased GDMT withdrawal in patients with HF with improved ejection fraction (HFimpEF) following successful AF catheter ablation.

## Methods

### Study Design and Population

This open-label, parallel-group pilot randomized clinical trial was conducted between April 13, 2023, and September 19, 2024, at Beijing Anzhen Hospital, China, to evaluate the feasibility and safety of phased withdrawal of GDMT in patients with suspected AMC. The trial protocol for this study is in Supplement 1. The study conformed to the Declaration of Helsinki^9^ and was approved by the Human Research Ethics Committee of Beijing Anzhen Hospital. Written informed consent was obtained from all participants. The study followed the Consolidated Standards of Reporting Trials (CONSORT) reporting guideline.

Inclusion criteria were: (1) aged 18 to 80 years; (2) 3 months’ post–radiofrequency catheter ablation of AF or atrial flutter; (3) LVEF 45% or less determined by echocardiography within 12 months before ablation; (4) current LVEF 55% or more and normal LV end-diastolic diameter (LVEDD), as assessed by echocardiography; (5) current N-terminal pro–brain natriuretic peptide (NT-proBNP) levels less than 250 ng/L; (6) current absence of symptoms and signs of HF; (7) currently treated with at least 1 GDMT; and (8) written informed consent.

Exclusion criteria were: (1) other suspected cardiomyopathy; (2) diagnosis of HF prior to AF or atrial flutter; (3) atrial tachycardia recurrence at 3 months after ablation (atrial tachyarrhythmia lasting >30 seconds); (4) premature ventricular contraction burden more than 10%, ventricular tachycardia, atrioventricular, or atrioventricular nodal reentry tachycardia; (5) 50% or more stenosis in any coronary artery; (6) uncontrolled hypertension (blood pressure >160/100 mm Hg); (7) moderate or severe valvular disease; (8) estimated glomerular filtration rate less than 30 mL/min/1.73 m^2^; (9) planned heart surgery within 1 year; (10) pregnancy or breastfeeding; or (11) life expectancy less than 1 year.

### Study Protocol

At 3 months after ablation, eligible patients were randomized 1:1 to phased GDMT withdrawal or continuation using computer-generated sequences via SAS software, version 9.4 (SAS Institute Inc) with enrolling physicians (Sitong Li and Y.S.) blinded to allocation.

In the GDMT withdrawal group, medications were reduced or discontinued stepwise every 2 weeks, with 1 medication adjusted at a time. For patients treated with more than 20 mg spironolactone, more than 10 mg finerenone, more than 40 mg furosemide, or more than 25% of an angiotensin receptor neprilysin inhibitor (ARNi) or a renin angiotensin system inhibitor (RASi) or β-blocker target doses, dosages were reduced by 50% every 2 weeks. Medication doses at or below these thresholds and sodium-glucose cotransporter-2 (SGLT2) inhibitors were discontinued immediately. The withdrawal sequence was loop diuretics, mineralocorticoid receptor antagonists (MRAs), β-blockers, ARNis or RASis, and SGLT2 inhibitors. Calcium channel blockers were used as substitutes for hypertension, and diabetes therapy was adjusted to avoid SGLT2 inhibitors. GDMT was reinitiated if the primary end point occurred. If patients continued to have elevated blood pressure, ARNis or RASis or β-blockers could be reinitiated; if elevated blood glucose persisted, SGLT2 inhibitors could be reinitiated. In the GDMT continuation group, GDMT type and dosage were maintained throughout follow-up.

Patients were followed up for 6 months. AF recurrence was managed according to guidelines and did not alter the withdrawal strategy. Patients underwent clinical assessment, NT-proBNP analysis, electrocardiography, 24-hour Holter monitoring, echocardiography, and cardiac magnetic resonance (CMR) imaging and were given the Kansas City Cardiomyopathy Questionnaire-12 (KCCQ-12), in which scores range from 0 to 100, with higher scores indicating better health status, at baseline (3 months after ablation, at the time of randomization). Follow-up visits were conducted monthly to document medications, NT-proBNP levels, echocardiography, and adverse drug events. CMR and KCCQ-12 assessments were conducted at 6 months. Imaging evaluators (H.W. and J.H.) were blinded to allocation. Details are in Supplement 1.

### Ablation Strategies and Antiarrhythmic Drug Use

All ablation procedures were performed using radiofrequency energy. Circumferential pulmonary vein isolation was routinely conducted for patients with paroxysmal AF. For those with persistent AF, the ablation strategy was most commonly following 2C3L (circumferential pulmonary vein isolation with 3 linear ablation lines: the left atrial roof line, mitral isthmus line, and tricuspid isthmus line) ablation with ethanol infusion into the vein of Marshall.^10^ Ablation strategy was determined at the operators’ (X.G., S.Z., X.L., N.L., Songnan Li, R.T., D.L., C.S., and C.M.) discretion (eTable 1 in Supplement 2).

Antiarrhythmic drugs were prescribed as needed during the 3-month blanking period. During the 3-month blanking period, 33 patients received amiodarone, 3 received propafenone, and the remaining 11 received no antiarrhythmic drug therapy. Antiarrhythmic drugs were discontinued at randomization.

### End Points

The primary end point was HF deterioration within 6 months, defined as meeting any of the following: more than a 10% decrease in LVEF to less than 55%, more than a 10% increase in LVEDD and to above the normal range (both assessed by echocardiography), a 2-fold rise in NT-proBNP levels and to more than 400 ng/L, or worsening HF signs and/or symptoms, as adjudicated by the research team (X.G., Songnan Li, and C.S.). We used the same thresholds as prior trials, such as TRED-HF^11^ and CATHEDRAL-HF,^12^ and other observational studies^13,14^ to better facilitate comparison of outcomes across studies. 

A key secondary outcome was a composite of cardiovascular death, hospitalization for HF, nonfatal stroke, or nonfatal myocardial infarction. Additional secondary end points included 6-month changes after randomization in LVEF and LVEDD (both assessed by echocardiography); CMR-based parameters including LVEF, LVED volume index (LVEDVi), LV global longitudinal strain (LV-GLS), and percentage of late gadolinium enhancement (LGE); and NT-proBNP level, KCCQ-12 score, heart rate, systolic blood pressure, and diastolic blood pressure. Other secondary outcomes included recurrence of atrial tachyarrhythmia, atrial tachyarrhythmia burden, and adverse drug events (Supplement 1). Atrial tachyarrhythmia burden was defined as the total time spent in atrial tachyarrhythmia during the Holter monitoring period, expressed as a percentage of the total monitored time.

### Statistical Analysis

This pilot trial aimed to assess the feasibility of GDMT withdrawal and inform the design of future larger trials, assuming most patients could safely withdraw GDMT without HF deterioration. As the pilot trial was not powered to detect differences in outcomes, a target sample size of 50 participants (25 per group) was chosen, based on the TRED-HF pilot trial, which enrolled 51 participants to compare GDMT withdrawal with continuation in patients with recovered dilated cardiomyopathy.^11^ Baseline characteristics were presented as median (IQR) for continuous variables and as number (percentage) for categorical variables. Analyses were conducted in the modified intention-to-treat population, which consisted of all randomized participants who had at least 1 postrandomization follow-up assessment. The primary end point was compared using the χ^2^ or Fisher exact test. Secondary end points were compared using the Wilcoxon rank sum test for non–normally distributed continuous variables, the *t* test for normally distributed continuous variables, and the χ^2^ or Fisher exact test for categorical variables. A 2-sided *P* < .05 was considered statistically significant. Statistical analyses were performed using R software, version 4.3.1 (R Project for Statistical Computing).

## Results

### Baseline Characteristics

**Figure 1.**
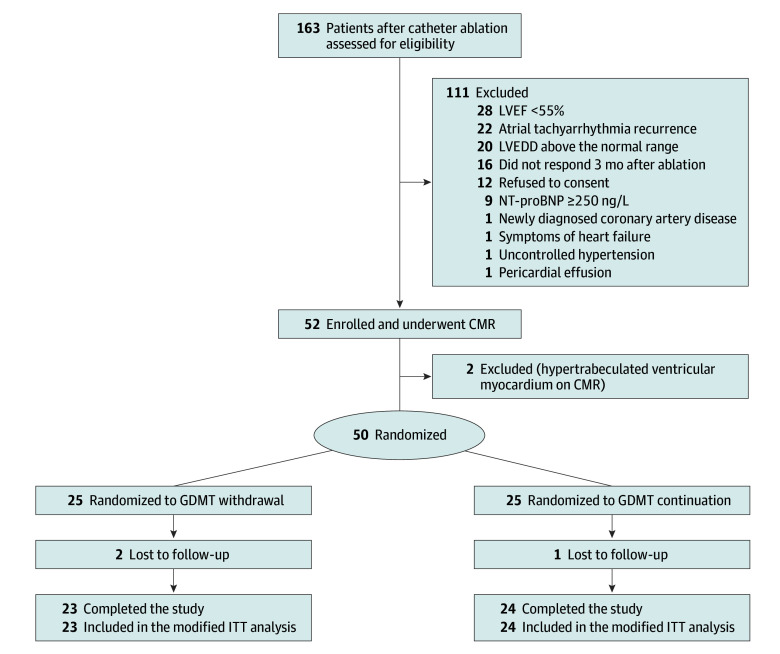
CONSORT Flow Diagram of Participant Flow Through Trial Screening log was not systematically collected. CMR indicates cardiac magnetic resonance; GDMT, guideline-directed medical therapy; ITT, intention-to-treat; LVEDD, left ventricular end-diastolic diameter; LVEF, LV ejection fraction; NT-proBNP, N-terminal pro–brain natriuretic peptide.

There were 50 eligible patients who were randomly assigned in a 1:1 ratio. Two patients in the GDMT withdrawal group and 1 in the GDMT continuation group were lost to follow-up. The study flowchart is presented in Figure 1. Among the 47 patients included in the final analysis, 10 (21.3%) were female, and 37 (78.7%) were male; the median (IQR) age was 56.0 (48.0-60.5) years. The lowest median (IQR) LVEF since the HF diagnosis was 38.0% (29.5%-41.0%). Patients were randomized at a median (IQR) of 98.0 (94.5-105.5) days after ablation. At randomization, the median (IQR) LVEF was 62.0% (59.5%-65.0%), and median (IQR) LVEDD was 49.0 (45.5-51.0) mm. Overall, 45 patients (95.7%) were treated with ARNis, 38 (80.9%) with β-blockers, 35 (74.5%) with MRAs, and 40 (85.1%) with SGLT2 inhibitors. CMR variables were comparable between groups (Table 1).

**Table 1.** Baseline Characteristics of Patients

Characteristic	Patients, No. (%)^a^	
	GDMT withdrawal (n = 23)	GDMT continuation (n = 24)
Age, median (IQR), y	56.0 (45.0 to 61.0)	56.0 (52.0 to 60.2)
Sex		
Female	5 (21.7)	5 (20.8)
Male	18 (78.3)	19 (79.2)
Body mass index, median (IQR)^b^	26.9 (24.5 to 30.1)	25.4 (23.3 to 27.4)
Type of AF or AFL		
Persistent AF	15 (65.2)	22 (91.7)
Paroxysmal AF	4 (17.4)	0
AFL	2 (8.7)	2 (8.3)
AF and AFL	2 (8.7)	0
Prior ablation	2 (8.7)	2 (8.3)
Medical history		
Hypertension	7 (30.4)	10 (41.7)
Diabetes	3 (13.0)	5 (20.8)
Stroke, TIA, or systemic embolism	1 (4.3)	4 (16.7)
Vascular disease	0	2 (8.3)
CHA_2_DS_2_-VA score, median (IQR)^c^	1.0 (1.0 to 2.0)	2.0 (1.0 to 2.0)
Time since AF diagnosis, median (IQR), mo	5.0 (4.0 to 27.0)	9.5 (5.9 to 15.0)
Time since HF diagnosis, median (IQR), mo	5.0 (3.5 to 5.8)	5.8 (3.5 to 8.2)
Lowest LVEF since HF diagnosis, median (IQR), %	38.0 (30.5 to 40.0)	36.0 (29.8 to 42.2)
Preablation LVEF, median (IQR), %	44.0 (38.5 to 52.5)	45.0 (40.0 to 55.0)
Preablation heart rate, median (IQR), bpm	101.0 (79.0 to 134.0)	105.5 (87.8 to 118.0)
Currently smoking	1 (4.0)	2 (8.0)
Current alcohol consumption	0	2 (8.0)
GDMT medication		
ARNi	23 (100.0)	22 (91.7)
β-Blocker	18 (78.3)	20 (83.3)
MRA	19 (82.6)	16 (66.7)
SGLT2 inhibitor	20 (87.0)	20 (83.3)
No. of GDMT medications, median (IQR)	4 (3 to 4)	4 (3 to 4)
Oral anticoagulants	19 (82.6)	17 (70.8)
Statins	9 (39.1)	10 (41.7)
Systolic blood pressure, median (IQR), mm Hg	118.0 (108.5 to 121.0)	108.0 (101.8 to 123.0)
Diastolic blood pressure, median (IQR), mm Hg	73.0 (68.0 to 81.5)	71.5 (64.0 to 76.0)
Heart rate, median (IQR), bpm	70.0 (64.0 to 74.0)	68.0 (64.5 to 71.5)
NT-proBNP level, median (IQR), pg/mL	39.0 (32.1 to 74.2)	66.6 (42.4 to 111.5)
eGFR, median (IQR), mL/min/1.73 m^2^	90.3 (78.7 to 96.6)	89.3 (79.5 to 94.3)
Echocardiographic variables, median (IQR)		
LVEF, %	61.0 (58.0 to 65.0)	62.5 (60.0 to 65.0)
LVEDD, mm	49.0 (47.0 to 51.0)	48.0 (45.0 to 50.0)
Left atrial diameter, mm	39.0 (35.0 to 41.0)	37.0 (35.0 to 39.2)
CMR variables, median (IQR)		
LVEF, %	57.4 (52.8 to 60.7)	56.6 (54.0 to 61.0)
LVEDVi, mL/m^2^	74.8 (64.7 to 85.4)	76.9 (66.6 to 80.3)
LV mass index, g/m^2^	52.7 (45.7 to 55.9)	50.7 (43.2 to 56.2)
LAVi, mL/m^2^	38.0 (31.5 to 43.0)	34.6 (28.0 to 40.0)
LV global longitudinal strain	−0.14 (−0.16 to –0.12)	−0.15 (−0.16 to –0.13)
Late gadolinium enhancement, %	7.3 (0 to 11.9)	9.3 (0 to 10.9)
KCCQ-12 score, median (IQR)^d^	100.0 (98.2 to 100.0)	100.0 (99.7 to 100.0)

Abbreviations: AF, atrial fibrillation; AFL, atrial flutter; ARNi, angiotensin receptor-neprilysin inhibitor; bpm, beats per minute; CHA_2_DS_2_-VA, congestive heart failure, hypertension, aged 75 years or older, diabetes, stroke, vascular disease; CMR, cardiac magnetic resonance; eGFR, estimated glomerular filtration rate; GDMT, guideline-directed medical therapy; HF, heart failure; KCCQ-12, Kansas City Cardiomyopathy Questionnaire-12; LAVi, left atrial volume index; LV, left ventricular; LVEDD, LV end-diastolic diameter; LVEDVi, LVED volume index; LVEF, LV ejection fraction; MRA, mineralocorticoid receptor antagonist; NT-proBNP, N-terminal pro–brain natriuretic peptide; SGLT2, sodium-glucose cotransporter-2; TIA, transient ischemic attack.

SI conversion factor: To convert NT-proBNP to ng/L, multiply by 1.0.

^a^ Data are reported as No. (%) unless otherwise indicated.

^b^ Calculated as weight in kilograms divided by height in meters squared.

^c^ Scores range from 0 to 8, with higher scores indicating greater thromboembolic risk.

^d^ Scores range from 0 to 100, with higher scores indicating better health status.

### Use of GDMT During Follow-Up

Use of HF medication at discharge (3 months before randomization) and at the end of the study (6 months after randomization) is summarized in eTable 2 in Supplement 2. Three patients in the GDMT withdrawal group restarted GDMT without meeting the primary end point: 1 restarted an ARNi, a beta-blocker, and an SGLT2 inhibitor due to a mean diastolic blood pressure of 95 mm Hg while treated with the nifedipine gastrointestinal therapeutic system, with uncontrolled blood glucose, and with a heart rate more than 100 beats per minute (bpm); another patient reinitiated an ARNi and a β-blocker because of an average blood pressure of 142 over 101 mm Hg while treated with the nifedipine gastrointestinal therapeutic system, with a heart rate more than 100 bpm; the third patient restarted a β-blocker for palpitations with a heart rate of 90 to 100 bpm. The proportions of GDMT use at randomization and during follow-up are shown in eFigure 1 in Supplement 2. At the 2-month follow-up, 20 patients (87.0%) in the GDMT withdrawal group were free from all GDMTs.

### Primary End Point

During the 6-month follow-up, 3 of 23 patients (13.0%) in the GDMT withdrawal group and none in the GDMT continuation group met the primary end point (*P* = .11). Among them, 2 patients met the NT-proBNP criterion (1632 pg/mL and 466 pg/mL), and 1 met the LVEF criterion (46%). All showed recovery of LVEF (from 46% to 56%) or NT-proBNP (from 466 pg/mL to 148 pg/mL and from 1632 pg/mL to 575 pg/mL) after GDMT reinitiation (eTable 3 in Supplement 2).

### Secondary End Points

**Figure 2.**
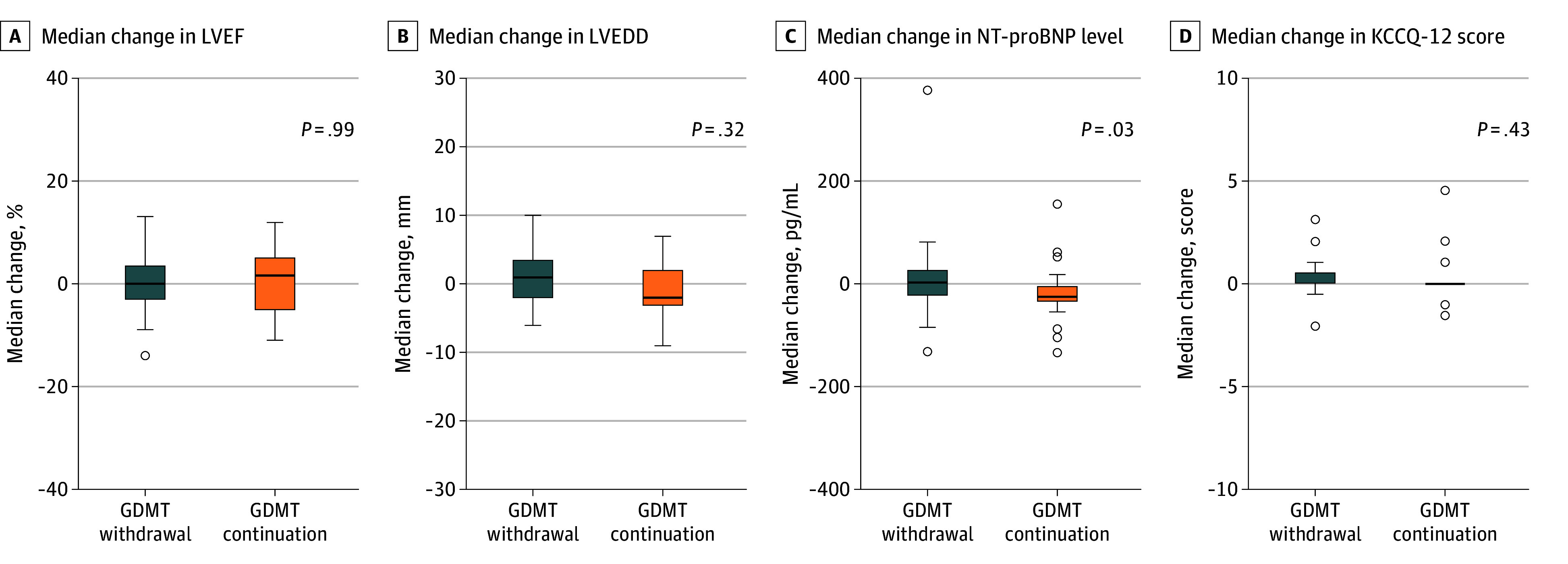
Box Plots of Changes of Echocardiographic Left Ventricular Ejection Fraction (LVEF), LV End-Diastolic Diameter (LVEDD), N-Terminal Pro–Brain Natriuretic Peptide (NT-proBNP) Level, and the Kansas City Cardiomyopathy Questionnaire-12 (KCCQ-12) Score From Baseline to 6 Months LVEF and LVEDD were analyzed with the *t* test, and NT-proBNP level and the KCCQ-12 score with the Wilcoxon rank sum test. The KCCQ-12 scores range from 0 to 100, with higher scores indicating better health status. GDMT indicates guideline-directed medical therapy. Horizontal bars inside boxes indicate the median; outer horizontal box lines, IQR; whiskers, ranges; outlier circles, data more extreme than whiskers.

No cardiovascular death, HF hospitalization, nonfatal stroke, or nonfatal myocardial infarction occurred in either group. The normality of continuous variables is presented in eFigures 2 and 3 in Supplement 2. Median (IQR) changes from baseline to 6 months were comparable between groups for echocardiographic LVEF (0% [−3.0% to 3.5%] vs 1.5% [−5.0% to 5.0%]; *P* = .99), LVEDD (1.0 [−2.0 to 3.5] mm vs −2.0 [−3.0 to 2.0] mm; *P* = .32), and KCCQ-12 scores (0 [0 to 0.5] vs 0; *P* = .43) (Figure 2). A greater median (IQR) improvement of the NT-proBNP level was observed in the GDMT continuation group than in the withdrawal group (−25.7 [−33.6 to −6.7] pg/mL vs 2.7 [−21.4 to 24.2] pg/mL; *P* = .03) (Figure 2). Median (IQR) changes of systolic blood pressure (11.0 [7.0 to 15.0] mm Hg vs 4.0 [−3.2 to 9.2] mm Hg; *P* = .003) and diastolic blood pressure (8.0 [1.5 to 17.5] mm Hg vs 2.5 [−1.5 to 10.0] mm Hg; *P* = .02) were higher in the GDMT withdrawal group, while heart rate changes were similar (13.0 [7.5 to 16.5] bpm vs 8.0 [2.0 to 13.2] bpm; *P* = .07).

**Figure 3.**
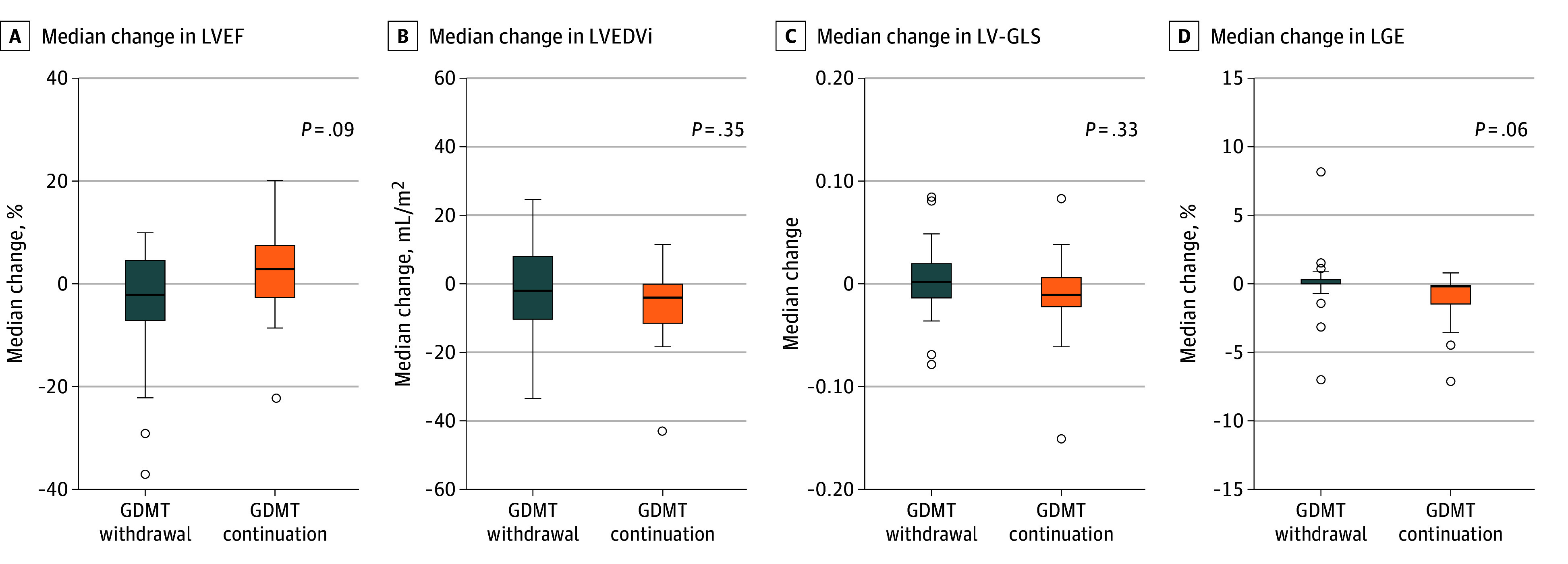
Box Plots of Changes of Left Ventricular Ejection Fraction (LVEF), LV End-Diastolic Volume Index (LVEDVi), LV Global Longitudinal Strain (LV-GLS), and Late Gadolinium Enhancement (LGE) on Cardiac Magnetic Resonance From Baseline to 6 Months Twenty-three patients were in each group. All variables were analyzed with the Wilcoxon rank sum test. GDMT indicates guideline-directed medical therapy. Horizontal bars inside boxes indicate the median; outer horizontal box lines, IQR; whiskers, ranges; outlier circles, data more extreme than whiskers.

One patient in the GDMT continuation group did not complete the follow-up CMR due to claustrophobia. Median (IQR) changes in CMR parameters over 6 months are displayed in Figure 3. No significant intergroup median (IQR) differences were found in LVEF (−2.1% [−7.2% to 4.6%] vs 2.9% [−2.6% to 7.4%]; *P* = .09), LVEDVi (−2.2 [−10.4 to 7.7] mL/m^2^ vs −4.1 [−11.6 to −0.1] mL/m^2^; *P* = .35), LV-GLS (0 [−0.01 to 0.02] vs −0.01 [−0.02 to 0.01]; *P* = .33), or percentage of LGE (0% [−0.10% to 0.30%] vs −0.10% [−1.45% to 0%]; *P* = .06) (Figure 3).

### Atrial Tachyarrhythmia Recurrence

All patients underwent a minimum of 24-hour Holter monitoring at 6 months after randomization. Additional opportunistic AF screening or symptom-triggered electrocardiogram recordings and Holter recordings during follow-up were also collected. Atrial tachyarrhythmia recurred in 3 patients in each group (13.0% vs 12.5%; *P* = .99). Among these, median (IQR) atrial arrhythmia burden was 7.0% (2.8%-100.0%) in the GDMT withdrawal group and 8.1% (4.6%-25.0%) in the GDMT continuation group. One patient in the GDMT continuation group underwent repeat ablation.

### Adverse Drug Events

Adverse drug events occurred in 5 of 24 patients in the continuation group and none in the withdrawal group (20.8% vs 0%; *P* = .050). Events included hypotension requiring an ARNi dose reduction (n = 3), sick sinus syndrome with the longest R-R interval of 5.8 seconds leading to β-blocker discontinuation (n = 1), and SGLT2 inhibitor withdrawal due to urogenital infection (n = 1). No kidney dysfunction, hyperkalemia, or diabetic ketoacidosis occurred in either group (Table 2). All other patients in the GDMT continuation group adhered to the protocol-prescribed GDMT throughout follow-up.

**Table 2.** Adverse Drug Events Between Groups

Adverse drug events	Patients, No. (%)	
	GDMT withdrawal (n = 23)	GDMT continuation (n = 24)
Composite events	0	5 (20.8)
Symptomatic hypotension	0	3 (12.5)
Kidney dysfunction	0	0
Hyperkalemia	0	0
Bradycardia	0	1 (4.2)
Diabetic ketoacidosis	0	0
Urogenital infection	0	1 (4.2)

Abbreviation: GDMT, guideline-directed medical therapy.

## Discussion

This randomized clinical trial assessed the feasibility and safety of GDMT withdrawal in patients with AF with HFimpEF after catheter ablation (patients with a high probability of AMC). Key findings were that (1) phased GDMT withdrawal may be considered in carefully selected patients; (2) no major adverse cardiovascular events occurred after GDMT withdrawal; (3) continued GDMT was associated with more adverse drug events; and (4) cardiac function improved after GDMT reinitiation in patients with HF deterioration.

Management of GDMT in patients with AF and improved LVEF remains challenging. While prolonged GDMT use may cause adverse effects and economic burden, withdrawal carries the risk of HF deterioration. The TRED-HF pilot randomized clinical trial showed a significantly higher HF relapse rate in recovered dilated cardiomyopathy after GDMT discontinuation vs continuation (45.7% vs 0%; *P* < .001).^11^ Accordingly, current guidelines recommend maintaining GDMT therapy in patients with HFimpEF.^5,15^ Moreover, recent clinical data in patients with AF and recovered LVEF showed that a RASi or an ARNi discontinuation was associated with a higher risk of cardiovascular mortality or HF hospitalization.^16^ However, recovery was defined only as an LVEF increase from less than 40% to 40% or more, without considering recovery duration or magnitude. Similarly, a single-center study of arrhythmia-induced cardiomyopathy (AIC) showed that maintaining RASis and β-blockers was associated with a significantly lower risk of HF relapse,^17^ but the lack of a defined recovery time frame limited differentiation from other cardiomyopathies.^18^ More recently, a multicenter study reported a modestly increased risk after GDMT withdrawal in patients with HFimpEF.^19^ However, HFimpEF is a heterogeneous population with diverse underlying mechanisms and prognoses.^20,21^ Most existing studies are observational and not specifically focused on AMC, limiting their applicability to these patients.

In clinical practice, the decision to discontinue GDMT in patients with AMC is often guided by physician judgment or patient preference. An observational study reported that 45% of patients with AF with recovered LVEF after ablation were no longer treated with HF medications at last follow-up, suggesting that withdrawal of HF treatment may be common and safe in selected patients.^8^ The 2017 Canadian HF guidelines proposed that patients with AIC with normalized LVEF and LV volume, New York Heart Association class I, and controlled tachycardia may be eligible for stepwise GDMT withdrawal after 6 to 12 months of therapy.^22^ The 2021 American Heart Association scientific statement on AF with HF with reduced ejection fraction acknowledged the ongoing debate regarding long-term HF therapy in AIC.^23^ A recent expert consensus highlighted that gradual GDMT discontinuation may be considered in recovered HF due to fully reversible etiologies through shared decision-making with close clinical, biomarker, and imaging monitoring.^24^ To date, the optimal duration of GDMT after LVEF recovery remains unclear due to limited evidence.^7^

In patients with AMC with normalized LVEF following AF rhythm control, the WITHDRAW-AF trial^25^ recently included 60 patients with an LVEF of 50% or more and sustained sinus rhythm for more than 6 months, randomized to a GDMT withdrawal or continuation group. Results showed that 90% of patients maintained an LVEF of 50% or more at 6 months after HF pharmacotherapy withdrawal, suggesting that GDMT discontinuation appeared safe in most selected patients.

Our study adds new evidence supporting close monitoring of patients undergoing phased GDMT withdrawal in carefully selected patients after catheter ablation, who were likely with AMC. We considered 3 months after ablation as the cardiac function recovery period, in line with recommendations that AIC be diagnosed within 1 to 6 months after arrhythmia elimination.^26^ We selected 3 months rather than a longer interval because earlier LVEF recovery may indicate more reversible myocardial injury, a better prognosis, and a higher likelihood of AMC.^2,20,26^ However, some observed changes may reflect a transient phase of reverse remodeling rather than fully sustained recovery. Notably, the WITHDRAW-AF trial, which included patients maintaining sinus rhythm for the preceding 6 months, reported results similar with ours.^25^ This suggests that using a 3-month vs a 6-month recovery period may have limited impact on HF deterioration after GDMT withdrawal.

Among patients with atrial tachyarrhythmia recurrence beyond the 3-month blanking period, the markedly reduced AF burden compared with preablation levels highlighted the efficacy of catheter ablation in maintaining rhythm control. Following HF treatment withdrawal, 2 patients had increased NT-proBNP levels, 1 had a decrease in LVEF percentage, and none showed evidence of LV dilation. Notably, 1 patient in the GDMT withdrawal group developed an elevated NT-proBNP level with normalized LV structure and systolic function after recurrent atrial flutter with a rapid ventricular rate, which was improved after rhythm control and GDMT reinitiation. The other 2 patients who met the primary end point also showed improvement in LVEF percentage or the NT-proBNP level after restarting HF therapy (eTable 3 in Supplement 2). These findings highlight the importance of regular rhythm monitoring, timely rhythm control, and prompt HF medication reinitiation in patients with recurrent atrial tachyarrhythmia.

Taken together, these observations also suggest that in patients with AF with HFimpEF, the definition of HF deterioration remains challenging, as AF recurrence itself may elevate NT-proBNP levels. However, relying solely on structural or functional parameters such as LVEF or LVEDD may be overly restrictive. In our study, only 1 patient experienced a decline in LVEF after GDMT withdrawal. Similar findings were reported in the WITHDRAW-AF trial, in which approximately 90% of patients maintained LVEF of 50% or more 6 months after HF therapy withdrawal.^25^ A 3-year follow-up study also indicated that arrhythmia-related hospitalizations and HF hospitalizations were uncommon after catheter ablation in patients with AF and HF with reduced ejection fraction.^27^ Importantly, no major adverse cardiovascular events occurred during follow-up in our study. Collectively, these findings suggest that surrogate end points may not adequately reflect clinically meaningful outcomes, while trials using event-driven end points as primary outcomes may be challenging to conduct in this setting. One-fifth of patients in the continued GDMT group had adverse drug events, suggesting that in carefully selected patients with recovered cardiac function, GDMT withdrawal may offer a favorable clinical net benefit. Future studies with larger sample sizes and event-driven end points are needed to determine whether GDMTs can be safely withdrawn and to identify appropriate candidates.

Regarding changes in CMR parameters between groups, no significant differences were observed in LVEF, LVEDVi, or LV-GLS, suggesting that GDMT withdrawal had no significant impact on LV structure and systolic and diastolic function. As a previous study showed similar LGE prevalence between patients with and without AIC,^28^ we did not exclude patients with LGE on CMR to enhance generalizability. Although the GDMT continuation group showed a slight decrease in the absolute percentage of LGE (−0.10%), this minimal change is unlikely to be clinically meaningful. Of the 3 patients who met the primary end point, 2 had a baseline presence of LGE, and 1 did not; none showed increased LGE following GDMT withdrawal (eTable 3 in Supplement 2). Whether the presence of LGE can predict HF deterioration after GDMT withdrawal warrants further investigation.

Our study has the strength to explore the feasibility of SGLT2 inhibitor withdrawal in the contemporary era of quadruple GDMT, with 85.1% of participants receiving SGLT2 inhibitors at baseline. In line with TRED-HF and WITHDRAW-AF, an ARNi or a RASi, a β-blocker, and an MRA were withdrawn biweekly until complete discontinuation. In patients with AF and HF, SGLT2 inhibitors were associated with a lower risk of AF recurrence after ablation.^29^ MRAs also reduced the risk of recurrent AF.^30^ Whether a slower de-escalation pace or a partial withdrawal strategy in patients with AMC could enhance safety and feasibility remains an important clinical question.

These findings were derived from patients with highly suspected AMC with recovered cardiac structure and function, an absence of HF symptoms, maintained sinus rhythm, and low NT-proBNP levels after AF catheter ablation. Given the relatively small sample size and the heterogeneity of populations with AF and HF, these findings should be interpreted carefully and not generalized to broader populations with AF or HF with reduced ejection fraction. Although follow-up was limited to 6 months, clinically relevant HF deterioration events were observed, underscoring the need for careful monitoring and larger studies with longer follow-up to better evaluate the safety of GDMT withdrawal in this specific patient population.

### Limitations

This study has several limitations. First, as a pilot randomized clinical trial with a limited sample size, potential baseline imbalances—such as the higher proportion of paroxysmal AF in the GDMT withdrawal group—may have influenced the observed HF deterioration outcomes despite randomization. However, patients with suspected AMC were difficult to enroll, as the diagnosis can only be established after rhythm control and exclusion of other HF causes. Second, follow-up was relatively short, and the 6-month assessment may primarily reflect early HF deterioration after GDMT withdrawal. Third, a substantial proportion of patients in the GDMT withdrawal group remained on certain components of GDMT, which may have influenced the observed treatment effects. Fourth, LVEF assessment by 2-dimensional echocardiography is subject to interobserver and intraobserver variability and may be overestimated compared with CMR. To address this, we adopted a historical LVEF less than 45% (rather than less than 40%) for inclusion, consistent with previous studies,^31,32,33^ and defined LVEF recovery as 55% or more, indicating an absolute improvement of 10% or more. CMR findings were consistent with echocardiographic assessments. Fifth, patients with irreversible atrial remodeling were not excluded due to the diagnostic challenges in this condition. Such patients may be less suitable for GDMT withdrawal. Sixth, genetic testing was not performed to explore the potential determinants of HF deterioration. Future long-term and large-sample trials with comprehensive clinical and biomarker assessments are needed to guide optimal therapeutic strategies.

## Conclusions

In this pilot randomized clinical trial of selected patients with AF with normalized cardiac function and sinus rhythm after catheter ablation, 3 of 23 patients (13%) with phased GDMT withdrawal had HF deterioration, compared with 0% among those who continued GDMT. Drug-related complications were more likely in those who continued GDMT. Further larger studies with longer follow-up are needed to determine whether GDMT can be safely discontinued in this population.

## Notes

Supplement 1.
Trial Protocol

Supplement 2. **eTable 1.** Ablation Strategies of Persistent Atrial Fibrillation **eTable 2.** Use of Heart Failure Medications at Discharge, Randomization, and 6-Month Follow-Up **eTable 3.** Characteristics of Patients Meeting the Primary End Point **eFigure 1.** Number of Patients Using GDMT at Randomization and Monthly Follow-Ups **eFigure 2.** Normal Q-Q Plots for Changes in Echocardiographic LVEF, LVEDD, NT-proBNP Level, and KCCQ-12 Score by GDMT Groups **eFigure 3.** Normal Q-Q Plots for Changes in LVEF, LVEDVi, LV-GLS, and LGE on CMR

Supplement 3.
Data Sharing Statement
