# Supplementary material for: Heart Failure Medication Withdrawal in Patients With Improved Cardiac Function After Atrial Fibrillation Ablation: The DEFINITION-AF Pilot Randomized Clinical Trial
Source: JAMA Netw Open. 2026 Jun 26;9(6):e2620145. doi: 10.1001/jamanetworkopen.2026.20145 (PMC13309871; doi:10.1001/jamanetworkopen.2026.20145)
Supplement: Supplement 1. — Trial Protocol [file jamanetwopen-e2620145-s001.pdf]

Study Protocol

With**D**rawal of pharmacological tr**E**atment for heart **F**ailure **I**n patie**N**ts with atrial fibrillation and heart failure with **I**mproved cardiac func**T**ion after ablat**ION** for **A**trial **F**ibrillation: an open-label, pilot, randomized trial **(DEFINITION-AF)**

Study Site: Beijing Anzhen Hospital, Capital Medical University
Principal Investigator: Caihua Sang, MD
Version and Date: V1.1 2023-03-22

Instructions for Protocol Completion:

1) For multicenter clinical trials, only the principal site shall be listed on the cover page. All other investigational sites shall be detailed within the body of the protocol.

2) For multicenter clinical trials, the coordinating investigator shall be listed as the principal investigator on the cover page.

3) Investigators shall organize and develop a scientifically sound and rational clinical trial protocol, taking into comprehensive account the study objectives, risk profiles, technical characteristics, and scope of application of the trial.

Table of Contents of the Study Protocol

List of Abbreviations 5
Protocol Summary 6
1. Information on the Clinical Research Site and Principal Investigator 9
2. Background of the Study 9
3. Study Objective 10
4. Study Design 10
4.1 Overall Design 10
4.2 Participant Selection 11
4.3 Study Outcomes 12
4.5 Study Flow 14
4.6 Imaging Assessment Methods 16
4.7 Bias Control 17
5. Statistical Methods 17
5.1 Sample Size Calculation 17
5.2 Principles of Statistical Analysis 18
5.3 Methods of Statistical Analysis 18
5.4 Handling of Missing and Aberrant Data 18
6. Data Management 19
6.1 Database Establishment and Data Collection 19
6.2 Raw Data Verification 19
6.3 Data Entry 19
6.4 Preservation of Data Management Documents and Data Transfer 19
7. Benefit and Risk Analysis 19
7.1 Potential Benefits 19
7.2 Potential Risks 19
8. Quality Control of the Clinical Trial 19
8.1 Training of Investigators and Research Assistants 19
8.2 Quality Control of Imaging Examinations 20
8.3 Measures to Improve Participant Compliance 20
9. Ethical Considerations and Informed Consent in the Clinical Trial 20
9.1 Ethical Considerations 20
9.2 Informed Consent Process 21
10. Regulations for Reporting Adverse Events 21

10.1 Definition and Reporting of Adverse Events 21
10.2 Serious Adverse Events 23
10.3 Reporting Procedures 23
11. Protocol Deviations and Amendments 23
11.1 Protocol Deviations 23
11.2 Measures to Control Protocol Deviations 23
11.3 Protocol Modification 24
12. Direct Access to Source Data and Documents 24
13. Contents of the Clinical Trial Report 25
14. Confidentiality Principles 25
15. Reference 25

List of Abbreviations

Abbreviation	English
ACEI	angiotensin-converting enzyme inhibitors
AMC	AF-mediated cardiomyopathy
ARB	angiotensin receptor blockers
ARNI	angiotensin receptorneprilysin inhibitors
β-b	β-blocker
CMR	cardiac magnetic resonance
CRF	case report form
CTCAE	events
GDMT	guideline-directed medical therapy
GLP-1	glucagon-like peptide-1
KCCQ	Kansas City Cardiomyopathy Questionnaire
LAVi	left atrial volume index
LGE	late gadolinium enhancement
LVEDD	left ventricular end-diastolic diameter
LVEDVi	left ventricular end-diastolic volume index
LVEF	left ventricular ejection fraction
LV-GLS	left ventricular global longitudinal strain
MRA	mineralocorticoid receptor antagonists
NT-proBNP	N-terminal pro-B type natriuretic peptide
SGLT2i	sodium-glucose cotransporter-2 inhibitor

Protocol Summary

**Study Title**	Withdrawal of pharmacological treatment for heart failure in patients with atrial fibrillation and heart failure with improved cardiac function after ablation for Atrial Fibrillation: an open-label, pilot, randomized trial (DEFINITION-AF)
**Study Design**	Prospective, single-center, open-label, randomized, controlled trial
**Objectives**	To evaluate the feasibility and safety of withdrawal of heart failure (HF) medications versus continued HF pharmacotherapy in patients with suspected atrial fibrillation-mediated cardiomyopathy (AMC) following successful catheter ablation.
**Intervention Group**	Withdrawal of HF Medications
**Control Group**	Continuation of HF Medications
**Methods**	Patients who have signed the Institutional Review Board (IRB) / Ethics Committee (EC)-approved Informed Consent Form (ICF) and are judged by the investigator to meet all inclusion and exclusion criteria will be enrolled into the study. Following enrollment, subjects will be randomized in a 1:1 ratio to either the intervention group or the control group. The intervention group will undergo stepwise withdrawal of HF medications, while the control group will continue the HF medications. Patients will be followed for 6 months to compare the efficacy and safety between the two groups.
**Sample Size**	This study requires the enrollment of 50 patients, with 25 patients allocated to each group.
**Inclusion and Exclusion Criteria**	1. Inclusion Criteria<br>1) Age 18 to 80 years old;<br>2) Having undergone catheter ablation for AF or atrial flutter (AFL) 3 months (±1 month) prior to enrollment;<br>3) Pre-ablation LVEF ≤45% as assessed by echocardiography, and tolerance of the ablation procedure with or without HF medications;<br>4) Taking GDMT post-ablation;

	5) Current LVEF ≥ 55% and LVEDD within the normal range, both assessed by echocardiography;<br>6) Current NT-proBNP <250ng/L;<br>7) Currently free from signs and symptoms related to HF;<br>8) Currently taking at least one of the following medications for HF: loop diuretics, ARNI/ACEi/ARB, β-b, MRA, or SGLT2i;<br>9) Able to provide written informed consent.<br>2. Exclusion Criteria<br>1) Coexisting other definite cardiomyopathy, such as ischemic cardiomyopathy, hypertrophic cardiomyopathy, restrictive cardiomyopathy, alcohol-induced cardiomyopathy, thyrotoxic cardiomyopathy, drug-induced cardiomyopathy, chemotherapy or cardiotoxic medications induced cardiomyopathy, myocarditis, Takotsubo cardiomyopathy, peripartum cardiomyopathy, et al.<br>2) A diagnosis of HF prior to AF or AFL;<br>3) AF/AFL/ Atrial tachycardia recurrence at 3 ± 1 months after catheter ablation;<br>4) Concomitant frequent premature ventricular contractions, ventricular tachycardia, or atrioventricular/atrioventricular nodal reentrant tachycardia;<br>5) Coronary artery disease definitively diagnosed by imaging;<br>6) Uncontrolled hypertension (blood pressure >160/100 mmHg);<br>7) Moderate-to-severe valvular heart disease;<br>8) eGFR <30 mL/min/1.73 m^2;<br>9) Planned cardiac surgery within 1 year;<br>10) Pregnancy or breasting;<br>11) Life expectancy <1 year (e.g., advanced malignant tumors).
**Primary Endpoint**	HF deterioration within 6 months;
**Secondary Endpoints**	1. Composite of cardiovascular death, hospitalization for HF, non-fatal stroke, or non-fatal myocardial infarction;<br>2. Changes from baseline to 6 months in the following parameters:<br>1) Echocardiography: LVEF, LVEDD;<br>2) CMR: LVEF, LVEDVi, LV-GLS, and percentage of LGE;

	3) NT-proBNP;<br>4) KCCQ-12 score;<br>5) Heart rate;<br>6) Systolic blood pressure;<br>7) Diastolic blood pressure.<br>3. Recurrence of atrial tachyarrhythmia and atrial tachyarrhythmia burden;<br>4. Adverse drug events, including symptomatic hypotension, renal dysfunction, hyperkalemia, bradycardia, diabetic ketoacidosis and urogenital infection.
**Statistical Analysis**	The study will follow a modified intention-to-treat approach. All patients, with the exception of those lost to follow-up, will be included in the statistical analysis according to their original randomization assignment.

1. Information on the Clinical Research Site and Principal Investigator

The clinical trial site for this study is Beijing Anzhen Hospital, Capital Medical University, and the principal investigator is Professor Caihua Sang.

2. Background of the Study

Atrial fibrillation (AF) is currently the most common cardiac arrhythmia. Its prevalence increases progressively with age. A large epidemiological survey conducted in China showed that among individuals aged 45 years and older, the age-standardized prevalence of AF is 1.8%, and among those aged 75 years and older, the prevalence exceeds 5%. It is estimated that approximately 7.9 million people aged ≥45 years in China have AF[1]. Heart failure (HF) is a terminal stage of many cardiovascular diseases, and its prevalence continues to rise. Recent epidemiological studies in China indicate that the prevalence of HF among individuals aged 35 years and older is approximately 1.3%, meaning that there are about 8.9 million HF patients in the country[2, 3].

Tachycardia-induced cardiomyopathy is the most important non-ischemic cause of heart failure with reduced ejection fraction (HFrEF) [4]. Following the publication of the CAMERA-MRI study [5], the 2020 European guidelines recommended catheter ablation—regardless of symptom status—for patients with a high suspicion of AF–mediated cardiomyopathy (AMC) to reverse left ventricular dysfunction, with a Class I (Level C) recommendation[6].

The CAMERA-MRI study enrolled 66 patients with persistent AF and idiopathic left ventricular ejection fraction (LVEF) ≤45%, who were randomized to catheter ablation or pharmacological rate control. After 6 months of follow-up, the catheter ablation group showed a significantly greater improvement in LVEF compared with the control group (18.4% vs. 4.4%, $P < 0.0001$) [5]. This indicates that AF-mediated left ventricular systolic dysfunction is potentially reversible through rhythm control [7]. The 2021 European HF guidelines further emphasize that some patients with HF may achieve complete recovery, including those with tachycardia-induced cardiomyopathy [8].

Although catheter ablation can improve cardiac function in patients with AMC, the optimal long-term HF medication strategy after cardiac function recovery remains unclear. Long-term medication adherence is often poor, and unnecessary drug use may lead to adverse effects such as hypotension, bradycardia, electrolyte disturbances, and hepatic or renal

dysfunction, as well as impose a substantial financial burden on patients and their families. The 2017 Canadian HF management guidelines state that patients with tachycardia-induced cardiomyopathy may be able to gradually withdraw guideline-directed medical therapy after 6-12 months of treatment if the following conditions are met: (1) normalization of LVEF and left ventricular volumes; (2) New York Heart Association (NYHA) class I functional status; and (3) adequate control of tachycardia. Long-term β-blocker therapy may still be required for rate control [9]. The 2021 AHA scientific statement on the management of AF with HFrEF notes that long-term management of HF medications in AMC remains controversial, although prolonged therapy may be beneficial for patients with persistent structural abnormalities[10].

Data from the China Atrial Fibrillation Registry suggest that in some patients who experienced improvement in cardiac function after AF catheter ablation did not develop HF symptoms after discontinuing HF medications, and their NT-proBNP, left ventricular end-diastolic diameter (LVEDD), and LVEF remained within normal ranges. We hypothesize that in patients with AMC who undergo catheter ablation and achieve recovery of cardiac function, HF medications may be safely withdrawn.

3. Study Objective

To compare the feasibility and safety of stepwise withdrawal of HF guideline-directed medical therapy (GDMT) versus continued GDMT therapy in patients with AF and improved cardiac function after successful catheter ablation (suspected AMC).

4. Study Design

4.1 Overall Design

This trial is a single-center, open-label, pilot, randomized controlled trial designed to assess the feasibility of conducting a large-scale investigation. The study will enroll patients with AF and HF who demonstrate improvement in cardiac function after catheter ablation.

Patients who sign an ethics committee–approved informed consent form and are deemed eligible for enrollment by the investigators may be included in the study. After enrollment, participants will be randomized 1:1 to phased GDMT withdrawal or continuation using computer-generated sequences via SAS software. The physicians responsible for enrollment and assignment of participants were blinded to the random allocation sequence. The experimental group will undergo stepwise withdrawal of GDMT, while the control group

will continue GDMT. All participants will be followed for 6 months to compare the safety and efficacy outcomes between the two groups.

4.2 Participant Selection

A total of 50 patients with AF and improved cardiac function after catheter ablation (suspected AMC) and are treated at Beijing Anzhen Hospital are planned for enrollment.

4.2.1 Inclusion Criteria

1) Age 18 to 80 years old;
2) Having undergone catheter ablation for AF or atrial flutter (AFL) 3 months (±1 month) prior to enrollment;
3) Pre-ablation LVEF ≤45% as assessed by echocardiography, and tolerance of the ablation procedure with or without HF medications;
4) Taking GDMT post-ablation;
5) Current LVEF ≥ 55% and LVEDD within the normal range, both assessed by echocardiography;
6) Current NT-proBNP <250ng/L;
7) Currently free from signs and symptoms related to HF;
8) Currently taking at least one of the following medications for HF: loop diuretics, ARNI/ACEi/ARB, β-b, MRA, or SGLT2i;
9) Able to provide written informed consent.

4.2.2 Exclusion Criteria

1) Coexisting other definite cardiomyopathy, such as ischemic cardiomyopathy, hypertrophic cardiomyopathy, restrictive cardiomyopathy, alcohol-induced cardiomyopathy, thyrotoxic cardiomyopathy, drug-induced cardiomyopathy, chemotherapy or cardiotoxic medications induced cardiomyopathy, myocarditis, Takotsubo cardiomyopathy, peripartum cardiomyopathy, et al.
2) A diagnosis of HF prior to AF or AFL;
3) AF/AFL/ Atrial tachycardia recurrence at 3 ± 1 months after catheter ablation;
4) Concomitant frequent premature ventricular contractions, ventricular tachycardia, or atrioventricular/atrioventricular nodal reentrant tachycardia;
5) Coronary artery disease definitively diagnosed by imaging;
6) Uncontrolled hypertension (blood pressure >160/100 mmHg);
7) Moderate-to-severe valvular heart disease;
8) eGFR <30 mL/min/1.73 m^2;
9) Planned cardiac surgery within 1 year;

10) Pregnancy or breasting;

11) Life expectancy <1 year (e.g., advanced malignant tumors).

4.2.3 Criteria and Procedures for Participant Withdrawal

4.2.3.1 Criteria for Withdrawal

All participants who have signed the informed consent form and successfully passed screening to enter the trial will be considered withdrawn if they discontinue the study at any time and for any reason before completing the observation period specified in the study protocol.

(1) Withdrawal Determined by the Investigator

a) Occurrence of a serious adverse event for which, in the investigator's judgment, trial participation should be discontinued;

b) Development of complications or special physiological changes during the trial that make continued participation inappropriate;

c) Poor adherence to the study protocol.

(2) Withdrawal Initiated by the Participant

a) The participant, for any reason, is unwilling or unable to continue the clinical trial and requests termination of participation;

b) The participant does not explicitly request withdrawal but discontinues treatment and follow-up, resulting in loss to follow-up.

(3) Handling of Withdrawn Cases

For all withdrawn cases, the status of clinical trial completion and the reasons for withdrawal should be documented. After a participant withdraws, the investigator should make every effort to contact the participant, inquire about the reason for withdrawal, record the most recent clinical symptoms, and complete any assessments that can still be performed. If withdrawal is due to an adverse event, the investigator should provide appropriate medical management based on the participant's condition.

When a participant or their legal guardian requests withdrawal from the trial, the investigator should ask whether previously collected data may still be used for the clinical study, and the response should be documented in the raw records.

4.3 Study Outcomes

4.3.1 Primary Endpoint

HF deterioration within 6 months.

Defined as meeting any of the following criteria:

(1) A decrease in LVEF on echocardiography by >10% in absolute value and to <55%;

(2) An increase in LVEDD on echocardiography by >10% and exceeding the normal range;

(3) A twofold rise in NT-proBNP and to >400 ng/L;

(4) None of the above criteria are met, but worsening HF signs and/or symptoms are present,, as adjudicated by the research team.

4.3.2 Secondary Endpoints

(1) Composite of cardiovascular death, hospitalization for HF, non-fatal stroke, or non-fatal myocardial infarction;

(2) Changes from baseline to 6 months in the following parameters:

a) Echocardiography: LVEF, LVEDD;

b) Cardiac magnetic resonance (CMR): LVEF, left ventricular end-diastolic volume index (LVEDVi), left ventricular global longitudinal strain (LV-GLS), and percentage of late gadolinium enhancement (LGE);

c) NT-proBNP;

d) KCCQ-12 score;

e) Heart rate;

f) Systolic blood pressure;

g) Diastolic blood pressure.

(3) Recurrence of atrial tachyarrhythmia and atrial tachyarrhythmia burden;

(4) Adverse drug events, including symptomatic hypotension, renal dysfunction, hyperkalemia, bradycardia, diabetic ketoacidosis and urogenital infection.

Definitions: 1) renal dysfunction: a decrease in the eGFR of ⩾50% or a decrease of >30 mL/min/1.73 m^2 from randomization to <60 mL/min/1.73 m^2; 2) hyperkalemia: serum potassium >5.5 mmol/L; and 3) bradycardia: heart rate at rest <40 beats/min.

4.4 Intervention

The intervention group will undergo a protocol-defined, stepwise withdrawal of HF medications, while the control group will continue GDMT for HF. Apart from the intervention under investigation, the management of AF/AFL and other non-HF comorbidities will be conducted in accordance with clinical guidelines for both groups. The investigators will evaluate and manage adverse drug events appropriately, which may include dose reduction or discontinuation of the offending medication.

4.4.1 Stepwise GDMT Withdrawal Group Protocol:

medications were reduced or discontinued stepwise every 2 weeks, with one medication adjusted at a time. For patients on >20 mg spironolactone, >10 mg finerenone, >40 mg furosemide, or >25% of ARNi/RASi or beta-blockers target doses, dosages were reduced by

50% every two weeks. Medication doses at or below these thresholds and SGLT2 inhibitors, were discontinued immediately. The withdrawal sequence was: loop diuretics, MRAs, beta-blockers, ARNi/RASi, and SGLT2 inhibitors. GDMT was reinitiated if the primary endpoint occurred.

4.4.2 Continuation Therapy Group Protocol:

GDMT type and dosage were maintained throughout follow-up

4.4.3 Specific Conditions:

1) Hypertension in GDMT withdrawal group: For newly developed hypertension or elevated blood pressure during follow-up, calcium channel blockers were used as substitutes. If patients continued to have elevated blood pressure while on calcium channel blockers, ARNi/RASi or beta-blockers could be reinitiated as appropriate.

2) Hypotension: Medication doses may be adjusted downward as clinically indicated.

3) Diabetes in GDMT withdrawal group: Diabetes therapy was adjusted to avoid SGLT2 inhibitors. If patients continued to have elevated blood glucose despite treatment with metformin combined with a dipeptidyl peptidase-4 inhibitor or an alpha-glucosidase inhibitor, SGLT2 inhibitors could be reinitiated.

4) Rapid Ventricular Rate: If ventricular rate exceeds 100 bpm in sinus rhythm or >110 bpm in AF/AFL or palpitation, β-b may be initiated or up-titrated as appropriate.

5) Others: Medication adjustments will be made based on guideline recommendations and clinical judgment.

4.4.4 Management of Atrial Tachyarrhythmia Recurrence

Throughout the 6-month follow-up period, if patients experience recurrence of atrial fibrillation or atrial flutter (AF/AFL), rhythm control strategies will be implemented in accordance with current clinical guidelines. These strategies include, but are not limited to: antiarrhythmic drug therapy, repeat catheter ablation, or cardioversion.

The occurrence of AF recurrence itself will not alter the GDMT withdrawal or continuation strategy. All patients will continue with their originally assigned protocol (GDMT withdrawal group or GDMT continuation group) according to randomization, with regular reassessment of cardiac function and clinical status.

4.5 Study Flow

4.5.1 Study Flowchart

**Visit**	**1**	**2**	**3**	**4**	**5**	**6**	**7**	**8**
**Case Report Form**	**Screening assessment**	**Baseline/ Randomization**						
**Time from Randomization**	**-1w~0w**	**0w**	**1m**	**2m**	**3m**	**4m**	**5m**	**6m**

Informed consent	▲							
Demographics	▲							
Medical history	▲							
Vital signs	▲	▲	▲	▲	▲	▲	▲	▲
Laboratory tests	▲	▲	▲	▲	▲	▲	▲	▲
24-hour Holter ECG	▲	▲	▲	▲	▲	▲	▲	▲
Echocardiography	▲	▲	▲	▲	▲	▲	▲	▲
CMR	▲							▲
KCCQ score	▲							▲
HF medications	▲	▲	▲	▲	▲	▲	▲	▲
Other cardiovascular medications	▲	▲	▲	▲	▲	▲	▲	▲

4.5.2 Study Implementation

4.5.2.1 Visit 1 (Screening Period: –1 week to 0 week)

(1) Basic Information

Obtain informed consent; record demographic data, medical history, allergy history, adverse events, and concomitant medications/treatments.

(2) Laboratory Tests

a) Complete blood count and C-reactive protein, including hemoglobin, hematocrit, red blood cell count, white blood cell count, platelet count, and C-reactive protein;
b) Blood biochemistry, including AST, ALT, total bilirubin, direct bilirubin, albumin, total protein, serum creatinine, LDL cholesterol, potassium, and sodium;
c) NT-proBNP;
d) Urinalysis: leukocytes, erythrocytes, and ketone bodies;
e) Stool tests: fecal occult blood and transferrin.

(3) Imaging Examinations

a) Echocardiography, including atrial and ventricular diameters, ventricular wall thickness, LVEF, and valvular regurgitation;
b) CMR, including LVEDVi, left ventricular end-systolic volume, left ventricular stroke volume, left ventricular cardiac output, left ventricular mass, LVEF, right ventricular end-diastolic diameter, right ventricular end-diastolic volume, right ventricular end-systolic volume, right ventricular stroke volume, right ventricular cardiac output, left atrial diameter, LAVi, right atrial diameter, delayed enhancement, extracellular volume, and global longitudinal strain;

(4) Other Assessments

Other evaluations include 24-hour Holter ECG and KCCQ score.

4.5.2.2 Visit2 (Baseline/Randomization 0 Week)

Investigators will enroll participants who have passed screening and meet the inclusion and exclusion criteria.

After enrollment, participants will be randomized in a 1:1 ratio using a random number table into either the experimental group or the control group. The experimental group will undergo stepwise withdrawal of HF medications, while the control group will continue standard HF therapy.

4.5.2.3 Visit 3-7 (Monthly Follow-up**)**

Participants will monitor blood pressure and heart rate daily. Monthly assessments will include NT-proBNP, liver and renal function tests with electrolytes, urinalysis, echocardiography, and 24-hour Holter ECG. At 6 months, CMR will be repeated along with KCCQ questionnaire evaluation. Echocardiography and 24-hour Holter ECG will also be performed, and current medication use will be recorded monthly.

4.5.2.4 Visit 8 (6-Month Post-Randomization)

Participants will continue daily monitoring of blood pressure and heart rate. Monthly assessments will include the aforementioned laboratory tests, echocardiography, and 24-hour Holter ECG, with current medication use recorded each month. At 6 months, CMR will be repeated, and the KCCQ questionnaire will be administered.

4.6 Imaging Assessment Methods

4.6.1 Echocardiography Protocol

Echocardiography was performed by experienced operators from the same echocardiography team using a commercially available system (Epic 7c, Philips Healthcare, Best, The Netherlands) equipped with a 3.5 MHz transducer for 2-dimensional imaging. All measurements were conducted in accordance with current guideline recommendations. LVEDD and left ventricular end-systolic diameters were manually traced at end-diastole and end-systole in both apical 4- and 2-chamber views. LVEF was calculated using the modified Simpson's biplane method. LA diameter was measured using the biplane method from apical views. Diastolic function was evaluated by measuring the mitral inflow E/A ratio and deceleration time of mitral inflow. In addition, tissue Doppler imaging was performed in the apical 4-chamber view. Peak systolic (S'), early diastolic (E') velocities of the medial and lateral mitral annulus, and the corresponding E/E' ratio were calculated. Sector width and

imaging depth were optimized individually to ensure the highest achievable frame rate and the optimal visualization.

4.6.2 Cardiac Magnetic Resonance Scan Protocol

CMR scans were performed on a 3-Tesla MR scanner (Ingenia CX, Philips Healthcare, Best, The Netherlands) with a 32-channel phased-array coil and a retrospective ECG gating. The CMR protocol comprised a standardized protocol including balanced steady-state free precession sequences for left ventricle cine images (short axis, 2, 3, 4 chambers), and Modified Look-locker inversion recovery (MOLLI) sequences for both pre- and post-contrast T1 mapping images, and late gadolinium enhancement images (LGE). LGE imaging was implemented 10 min after intravenous administration of contrast medium (0.2 mmol/kg; Magnevist gadopentetate dimeglumine, Bayer Healthcare, Bayer, Berlin, Germany) using a phase-sensitive inversion-recovery (PSIR) TFE sequence. General CMR analyses (cardiac function, myocardial strain, extent and distribution of LGE, T1 mapping) were conducted using the CVI 42 software (Circle Cardiovascular Imaging, Calgary, Canada) by a radiologist who was blinded to treatment allocation.

4.7 Bias Control

4.7.1 Prospective Trial

The prospective design of this trial, with a pre-specified study population and standardized protocol, inherently mitigates biases common in retrospective studies, such as recall and selection bias. The analysis will follow a modified intention-to-treat (ITT) principle, including all randomized participants who are not lost to follow-up in their originally assigned groups, thereby minimizing attrition bias. This approach helps ensure the robustness and credibility of the trial's findings.

4.7.2 Investigator Training

Before the initiation of the clinical trial, monitors will provide training to investigators on the study protocol, ensuring that they understand and are familiar with the medication regimen and follow-up procedures.

4.7.3. Standardized Implementation of Inclusion and Exclusion Criteria

Participants will be screened according to the trial's inclusion and exclusion criteria to minimize selection bias.

5. Statistical Methods

5.1 Sample Size Calculation

This study is designed to explore the feasibility of a future large-scale trial. The sample size is set at 50 participants, based on a 2019 Lancet study investigating withdrawal of HF medications in patients with dilated cardiomyopathy[11].

5.2 Principles of Statistical Analysis

Statistical analysis will be performed according to the modified intention-to-treat (ITT) approach.

5.3 Methods of Statistical Analysis

5.3.1 Statistical Analysis Software

R version 4.2.1 or higher will be used for all analyses.

5.3.2 General Principles

Descriptive Statistics: For quantitative variables, the number of observations, mean, standard deviation, median, interquartile range, minimum, and maximum will be reported. For categorical variables, counts and percentages for each category will be presented.

Inferential Statistics: Unless otherwise specified, all statistical tests will be two-sided, and a P-value ≤ 0.05 (two-sided) will be considered statistically significant. The primary endpoint was compared using the chi-squared or Fisher's exact test. Secondary endpoints were compared using Wilcoxon rank-sum test for continuous variables, and chi-squared or Fisher's exact test for categorical variables.

5.4 Handling of Missing and Aberrant Data

A statistical analysis plan will be developed before the trial begins. After database lock, statistical analyses will be conducted according to the plan, and a statistical analysis report will be generated based on the results.

5.4.1 Handling of Missing Data: Missing data will not be imputed; analyses will be performed using the available data.

5.4.2 Handling of Erroneous Data: During data management, quality control will be performed on the database. Identified erroneous data will be queried to the investigators via a data query form. Corrections will be made based on the investigators' written responses. The database will only be locked after all erroneous data have been corrected.

5.4.3 Handling of Implausible Data: During data management, logical checks will be performed on the database. Implausible data will be queried to the investigators via a data query form. Adjustments will be made based on the investigators' written responses. The database will only be locked after all implausible data have been resolved.

6. Data Management

6.1 Database Establishment and Data Collection

The trial will collect all study data using a standardized electronic data-entry system according to the protocol. All variables, form structures, and data-entry rules are predefined, and every recorded item must be verifiable against source documents. The study database will be built and maintained by trained data-management staff to ensure data quality.

6.2 Raw Data Verification

Investigators will confirm that all electronically recorded data match the source documents. Any inconsistencies will trigger data queries, which site staff will resolve by checking the original records and updating the database accordingly to ensure accuracy and completeness.

6.3 Data Entry

Data will be entered by two data entry personnel independently in duplicate. After consistency checks, any discrepancies will be verified and corrected. Once all data are reconciled, the database will be submitted to the data management manager for final review.

6.4 Preservation of Data Management Documents and Data Transfer

The data management manager will retain all data management–related documents as required and will transfer the locked database and related materials to the statistician for statistical analysis.

7. Benefit and Risk Analysis

7.1 Potential Benefits

Both treatment strategies in this study have their respective advantages. Regardless of group assignment, participants will receive close monitoring by physicians, providing individualized treatment and medical guidance. The costs of CMR and echocardiography at the 6-month follow-up will be covered by the investigators for all participants.

7.2 Potential Risks

Adverse events may occur during participation in this study, although they may not be related to the clinical trial. Participation may also result in no improvement in the participant's condition.

8. Quality Control of the Clinical Trial

8.1 Training of Investigators and Research Assistants

To ensure trial quality, all investigators and research assistants must undergo training before the trial begins. Training will cover, but is not limited to, the study protocol, adjustment of

HF medications, and recording and reporting of adverse events. Continuous training should be provided during the trial as needed. When significant updates to the protocol or related documents occur, retraining of the project team and investigator team is required. For trial personnel joining after the initiation meeting, complete training must be conducted before they engage in any trial activities. All completed trainings must be documented with signed training records.

8.2 Quality Control of Imaging Examinations

To ensure the quality of echocardiography and CMR, the same sonographer and CMR physician will perform all examinations on the same equipment for each patient, minimizing bias.

8.3 Measures to Improve Participant Compliance

Informed consent will be carefully implemented to ensure participants fully understand the trial requirements and cooperate with study procedures.

Investigators will provide close follow-up, monitoring symptoms, blood pressure, heart rate, medication adherence, and test results, and offer individualized medical advice. The costs of CMR and echocardiography at the 6-month follow-up will be covered by the investigators.

9. Ethical Considerations and Informed Consent in the Clinical Trial

9.1 Ethical Considerations

The clinical trial must comply with the Declaration of Helsinki and relevant national regulations and standards for clinical trials. The trial may only commence after approval of the study protocol by the ethics committee of the responsible clinical trial institution.

Before enrolling each participant, the investigator is responsible for providing a complete and comprehensive explanation in writing to the participant or their designated representative regarding the purpose, procedures, and potential risks of the trial. Participants must be informed of their right to withdraw from the trial at any time. For prospectively enrolled participants, a written informed consent form must be provided prior to enrollment, and the trial physician is responsible for ensuring that each participant has given informed consent before entering the study. The signed informed consent form must be retained as part of the clinical trial documentation.

If the informed consent form is revised during the trial, the revised version must first be approved by the ethics committee before implementation. Once approved, all participants whose follow-up is not yet completed must sign the revised informed consent form.

Before initiating the trial, investigators must submit the study protocol, informed consent form, and other relevant documents to the ethics committee of the hospital overseeing the

clinical trial. The trial may only begin after obtaining ethics committee approval. Any amendments to the study protocol must also be approved by the ethics committee before implementation. Serious adverse events occurring during the trial must be reported promptly in writing to the ethics committee.

9.2 Informed Consent Process

For prospectively enrolled participants, investigators are responsible for providing a complete and comprehensive written explanation of the trial's purpose, procedures, and potential risks to each participant or their legal guardian before enrollment. Participants must be informed of their right to withdraw from the trial at any time, and their personal information will be kept confidential. Each participant must receive a written informed consent form prior to enrollment. Investigators are responsible for obtaining the signed informed consent form before the participant enters the study and for retaining it in the trial records.

For participants who lack decision-making capacity, enrollment is permissible if the ethics committee gives preliminary approval and the investigator determines that participation is in the participant's best interest. In such cases, the participant's legal guardian must sign and date the informed consent form before trial initiation.

If the participant or their legal guardian is unable to read, a witness must be present during the informed consent process. The informed consent form must be thoroughly explained, and the witness must confirm that the written form is consistent with the oral explanation. The participant or guardian provides verbal consent, after which the witness signs and dates the consent form. The witness's signature and the investigator's signature must be completed on the same day.

10. Regulations for Reporting Adverse Events

10.1 Definition and Reporting of Adverse Events

10.1.1 Definition

An adverse event (AE) is any unfavorable medical occurrence that arises during the clinical trial, regardless of whether it is related to the study.

10.1.2 Reporting Regulations

1) Grading of Adverse Event Severity

The severity of adverse events will be graded based on the Common Terminology Criteria for Adverse Events (CTCAE) version 5.0. The criteria for evaluating adverse events are shown in Table 11-1.

Table 10-1. Adverse Event Evaluation Criteria

Severity Grading	Criteria[1]
Grade 1	Mild; asymptomatic or minimal symptoms; only detectable clinically or by diagnostic tests; no treatment required.
Grade 2	Moderate; requires minor, local, or non-invasive intervention; instrumental activities of daily living limited [2].
Grade 3	Severe or medically significant but not immediately life-threatening; results in hospitalization or prolongation of hospitalization; causes disability; basic activities of daily living limited[3].
Grade 4	Life-threatening; requires urgent intervention.
Grade 5	Death related to the adverse event.

Notes:

[1] In the criteria, ";" denotes "or."

[2] Instrumental activities of daily living refer to cooking, shopping for clothes, using the telephone, managing finances, etc.

[3] Basic activities of daily living refer to bathing, dressing/undressing, eating, toileting, taking medications, and not being bedridden long-term.

2) Adverse Event Documentation and Reporting

Adverse Events: Record any adverse events that occurred since the previous visit, including cardiovascular death and other significant adverse events, using the adverse event reporting form.

Patient Death (if applicable): If a participant dies before a scheduled visit, the event must be reported immediately to the sponsor.

3) Management of Adverse Events

a) General Principles

Once an adverse event occurs, investigators must ensure that participants receive adequate and timely treatment and management. All adverse events (AEs) and serious adverse events (SAEs) must be followed up until one of the following occurs:

i. The event resolves.
ii. The event stabilizes.
iii. The event returns to baseline if a baseline value is available and acceptable.
iv. No further information can be obtained (e.g., the participant or caregiver refuses to provide additional information despite documented follow-up efforts, resulting in loss to follow-up).

b) Potential Adverse Events and Management

Potential study-related adverse events include cardiovascular death, nonfatal stroke, nonfatal myocardial infarction, heart failure–related hospitalization, acute kidney injury, and others. Physicians should manage these events symptomatically, actively investigate the cause, implement appropriate treatments, and report according to protocol.

10.2 Serious Adverse Events

A serious adverse event (SAE) is defined as any event during the clinical trial that results in death or a significant deterioration in health, including: life-threatening conditions, fatal disease or injury, permanent structural or functional disability, hospitalization or prolonged hospitalization, medical intervention required to prevent permanent impairment of body structure or function, fetal distress, fetal death, or congenital anomalies or birth defects.

10.3 Reporting Procedures

All adverse events occurring during the clinical trial must be documented and analyzed by the investigator. A written report should be prepared, including the investigator's recommendations on whether to continue, suspend, or terminate the trial, and submitted to the ethics committee.

11. Protocol Deviations and Amendments

11.1 Protocol Deviations

The clinical trial must be conducted in accordance with the protocol approved by the ethics committee. Any intentional or unintentional departure from the approved protocol is considered a protocol deviation.

Protocol deviations can be classified by the responsible party: deviations due to noncompliance by the trial site, or deviations due to noncompliance by the participant. They can also be classified by severity: minor deviations and major deviations. Major deviations have one or more of the following characteristics: affect participant safety or rights; influence the participant's willingness to continue in the trial; affect the quality or integrity of the data. Minor deviations generally do not exhibit any of these three characteristics.

Minor deviations are usually reported periodically to the ethics committee, with investigators explaining the cause, impact, and corrective measures. Major deviations must be reported promptly.

For every protocol deviation, corrective or preventive measures should be implemented to rectify the error or prevent recurrence of similar deviations.

11.2 Measures to Control Protocol Deviations

11.2.1 Investigators

1) During the trial initiation and feasibility assessment stage, thoroughly discuss the protocol's implementability at the study site.
2) Investigators must explain to participants the importance of adhering to the protocol during the informed consent process.

3) Investigators must comply with the ethics committee–approved protocol. If a protocol deviation is recognized, it should be immediately documented and explained.

11.2.2 Participants

When re-signing the informed consent form, investigators should provide detailed explanations of the study protocol and requirements, including important assessments, follow-ups, and other precautions.

11.2.3 Retraining

Investigators must undergo retraining, potentially multiple times if necessary, in the following situations:

1) Misunderstanding of the protocol;
2) New investigators join the study;
3) Changes in the trial plan;
4) Reminders to investigators regarding protocol details.

11.2.4 Protocol Deviation Reporting

The principal investigator must report trial progress to the ethics committee on schedule and promptly report any events or protocol deviations affecting participant rights or safety.

In emergencies where deviations cannot be reported immediately, they should be reported in writing as soon as possible in accordance with relevant regulations.

During the trial, any amendments to the protocol or informed consent documents, or resumption of a suspended trial, must be approved in writing by the ethics committee before implementation.

Investigators must strictly follow the protocol and may not deviate or make substantive changes without ethics committee approval. However, in urgent situations where participants face direct danger, deviations may be implemented immediately, with written reports submitted afterward.

11.3 Protocol Modification

During the clinical trial, if investigators identify deficiencies in the protocol—such as inclusion/exclusion criteria that do not adequately cover the target population, difficulties in enrolling eligible participants, or sample size calculations based on parameter values inconsistent with clinical reality leading to insufficient sample size—the protocol should be revised. All amendments must be submitted to and approved by the ethics committee.

12. Direct Access to Source Data and Documents

Source Data refers to all original records of clinical findings, observations, and other activities in a medical device clinical trial, as well as their verified copies, which can be used to reconstruct and evaluate the clinical trial.

Source Documents include printed, visual, or electronic files that contain source data.

In this trial, the principal investigator and authorized investigators may access and generate inpatient medical records, participant informed consent forms, CRFs, and original case records in the HIS system.

Laboratory personnel and investigators may access and generate the test result reports they produce. Data management personnel may access and generate data management datasets. Statistical analysts may access and generate statistical analysis datasets. Access and editing rights for other source data shall be governed by relevant Chinese laws, regulations, and technical standards, with detailed procedures specified in the corresponding departmental SOPs.

13. Contents of the Clinical Trial Report

The clinical trial report should generally include the following: basic information of the medical device clinical trial, trial conduct, statistical analysis methods, trial results, adverse event reporting and management, analysis and discussion of trial results, clinical trial conclusions, ethical considerations, identified issues, and recommendations for improvement.

14. Confidentiality Principles

This trial protocol is confidential and is intended for use by medical experts involved in the trial, investigators participating in the study, trial-related personnel, the medical institutions conducting the trial, the ethics committee, and any contract research organizations or other entrusted institutions involved in the trial.

15. Reference

1. Du X, Guo L, Xia S, Du J, Anderson C, Arima H, Huffman M, Yuan Y, Zheng Y, Wu S, et al. Atrial fibrillation prevalence, awareness and management in a nationwide survey of adults in China. *Heart*. 2021;107:535-41. doi: 10.1136/heartjnl-2020-317915

2. Hao G, Wang X, Chen Z, Zhang L, Zhang Y, Wei B, Zheng C, Kang Y, Jiang L, Zhu Z, Zhang J, Wang Z and Gao R. Prevalence of heart failure and left ventricular dysfunction in

China: the China Hypertension Survey, 2012-2015. *Eur J Heart Fail*. 2019;21:1329-1337. doi: 10.1002/ejhf.1629

3. Metra M and Lucioli P. Corrigendum to 'Prevalence of heart failure and left ventricular dysfunction in China: the China Hypertension Survey, 2012-2015' [Eur J Heart Fail 2019;21:1329-1337]. *Eur J Heart Fail*. 2020;22:759. doi: 10.1002/ejhf.1808
4. Reddy YNV, Borlaug BA and Gersh BJ. Management of Atrial Fibrillation Across the Spectrum of Heart Failure With Preserved and Reduced Ejection Fraction. *Circulation*. 2022;146:339-357. doi: 10.1161/circulationaha.122.057444
5. Prabhu S, Taylor AJ, Costello BT, Kaye DM, McLellan AJA, Voskoboinik A, Sugumar H, Lockwood SM, Stokes MB, Pathik B, et al. Catheter Ablation Versus Medical Rate Control in Atrial Fibrillation and Systolic Dysfunction: The CAMERA-MRI Study. *J Am Coll Cardiol*. 2017;70:1949-1961. doi: 10.1016/j.jacc.2017.08.041
6. Hindricks G, Potpara T, Dagres N, Arbelo E, Bax JJ, Blomström-Lundqvist C, Boriani G, Castella M, Dan GA, Dilaveris PE, et al. 2020 ESC Guidelines for the diagnosis and management of atrial fibrillation developed in collaboration with the European Association for Cardio-Thoracic Surgery (EACTS): The Task Force for the diagnosis and management of atrial fibrillation of the European Society of Cardiology (ESC) Developed with the special contribution of the European Heart Rhythm Association (EHRA) of the ESC. *Eur Heart J*. 2021;42:373-498. doi: 10.1093/eurheartj/ehaa612
7. Pabel S and Sossalla S. Atrial fibrillation and heart failure: novel insights into the chicken and egg dilemma. *Eur Heart J*. 2022;43:3376-3378. doi: 10.1093/eurheartj/ehac257
8. McDonagh TA, Metra M, Adamo M, Gardner RS, Baumbach A, Böhm M, Burri H, Butler J, Čelutkienė J, Chioncel O, et al. 2021 ESC Guidelines for the diagnosis and treatment of acute and chronic heart failure. *Eur Heart J*. 2021;42:3599-3726. doi: 10.1093/eurheartj/ehab368
9. Ezekowitz JA, O'Meara E, McDonald MA, Abrams H, Chan M, Ducharme A, Giannetti N, Grzeslo A, Hamilton PG, Heckman GA, et al. 2017 Comprehensive Update of the Canadian Cardiovascular Society Guidelines for the Management of Heart Failure. *Can J Cardiol*. 2017;33:1342-1433. doi: 10.1016/j.cjca.2017.08.022
10. Gopinathannair R, Chen LY, Chung MK, Cornwell WK, Furie KL, Lakkireddy DR, Marrouche NF, Natale A, Olshansky B and Joglar JA. Managing Atrial Fibrillation in Patients With Heart Failure and Reduced Ejection Fraction: A Scientific Statement From the American Heart Association. *Circ Arrhythm Electrophysiol*. 2021;14:Hae0000000000000078. doi: 10.1161/hae.0000000000000078

11. Halliday BP, Wassall R, Lota AS, Khalique Z, Gregson J, Newsome S, Jackson R, Rahneva T, Wage R, Smith G, et al. Withdrawal of pharmacological treatment for heart failure in patients with recovered dilated cardiomyopathy (TRED-HF): an open-label, pilot, randomised trial. *Lancet*. 2019;393:61-73. doi: 10.1016/s0140-6736(18)32484-x
