# Supplementary material for: Heart Failure Medication Withdrawal in Patients With Improved Cardiac Function After Atrial Fibrillation Ablation: The DEFINITION-AF Pilot Randomized Clinical Trial
Source: JAMA Netw Open. 2026 Jun 26;9(6):e2620145. doi: 10.1001/jamanetworkopen.2026.20145 (PMC13309871; doi:10.1001/jamanetworkopen.2026.20145)
Supplement: Supplement 2. — eTable 1. Ablation Strategies of Persistent Atrial Fibrillation eTable 2. Use of Heart Failure Medications at Discharge, Randomization, and 6-Month Follow-Up eTable 3. Characteristics of Patients Meeting the Primary End Point eFigure 1. Number of Patients Using GDMT at Randomization and Monthly Follow-Ups eFigure 2. Normal Q-Q Plots for Changes in Echocardiographic LVEF, LVEDD, NT-proBNP Level, and KCCQ-12 Score by GDMT Groups eFigure 3. Normal Q-Q Plots for Changes in LVEF, LVEDVi, LV-GLS, and LGE on CMR [file jamanetwopen-e2620145-s002.pdf]

Supplemental Online Content

Li S, Sun Y, Lai Y, et al. Heart failure medication withdrawal in patients with improved cardiac function after atrial fibrillation ablation: the DEFINITION-AF pilot randomized clinical trial. *JAMA Netw Open*. 2026;9(6):e2620145.
doi:10.1001/jamanetworkopen.2026.20145

eTable 1. Ablation Strategies of Persistent Atrial Fibrillation
eTable 2. Use of Heart Failure Medications at Discharge, Randomization, and 6-Month Follow-Up
eTable 3. Characteristics of Patients Meeting the Primary End Point
eFigure 1. Number of Patients Using GDMT at Randomization and Monthly Follow-Ups
eFigure 2. Normal Q-Q Plots for Changes in Echocardiographic LVEF, LVEDD, NT-proBNP Level, and KCCQ-12 Score by GDMT Groups
eFigure 3. Normal Q-Q Plots for Changes in LVEF, LVEDVi, LV-GLS, and LGE on CMR

This supplemental material has been provided by the authors to give readers additional information about their work.

© 2026 Li S et al. *JAMA Network Open.*

eTable 1. Ablation Strategies of Persistent Atrial Fibrillation

**Ablation Strategies**	**Number (%)**
Pulmonary vein isolation	2 (5.4%)
‘2C3L’ ablation*	8 (21.6%)
‘2C3L’ ablation plus ethanol infusion into the vein of Marshall	19 (51.4%)
Pulmonary vein isolation plus linear ablation	6 (16.2%)
Pulmonary vein isolation plus posterior wall isolation or complex fractionated atrial electrogram ablation	2 (5.4%)

*‘2C3L’ ablation comprises pulmonary vein isolation, as well as roof line, mitral isthmus line, and cavotricuspid isthmus line ablations.

© 2026 Li S et al. *JAMA Network Open.*

eTable 2. Use of Heart Failure Medications at Discharge, Randomization, and 6-Month Follow-Up

**Heart failure medications, n (%)**	**GDMT withdrawal (n=23)**	**GDMT continuation (n=24)**
**At Discharge**		
ARNi	20 (87.0)	20 (83.3)
Beta-blocker	10 (43.5)	15 (62.5)
MRA	15 (65.2)	17 (70.8)
SGLT2 inhibitor	16 (69.6)	14 (58.3)
Loop diuretics	11 (47.8)	8 (33.3)
Vericiguat	1 (4.3)	1 (4.2)
Tolvaptan	0 (0.0)	1 (4.2)
**At Randomization**		
ARNi	23 (100.0)	22 (91.7)
Beta-blocker	18 (78.3)	20 (83.3)
MRA	19 (82.6)	16 (66.7)
SGLT2 inhibitor	20 (87.0)	20 (83.3)
**At 6-month Follow-up**		
ARNi	3 (13.0)	22 (91.7)
Beta-blocker	4 (17.4)	19 (79.2)
MRA	1 (4.3)	16 (66.7)
SGLT2 inhibitor	3 (13.0)	19 (79.2)

ARNi, angiotensin receptor-neprilysin inhibitor; GDMT, guideline-directed medical therapy; HF, heart failure; MRA, mineralocorticoid receptor antagonist; SGLT2, sodium-glucose cotransporter 2.

© 2026 Li S et al. *JAMA Network Open.*

eTable 3. Characteristics of Patients Meeting the Primary End Point

**No.**	**Age, years**	**Sex**	**Baseline**			**Endpoint**			**LGE on CMR**		**AFL recurrence**	**After GDMT re-initiation**		
			LVEF	LVEDD	NT-proBNP	LVEF	LVEDD	NT-proBNP	Baseline	6-m FU		LVEF	LVEDD	NT-proBNP
1	54	Male	58%	50 mm	89.9 pg/mL	62%	47 mm	466 pg/mL	12.9%	12.8%	No	60%	53 mm	148 pg/mL
2	67	Male	65%	51 mm	82.3 pg/mL	56%	50 mm	1632 pg/mL	11.6%	11.3%	Yes	66%	56 mm	575 pg/mL
3	67	Male	70%	52 mm	35.7 pg/mL	46%	51 mm	138.7 pg/mL	0%	0%	No	56%	53 mm	75.6 pg/mL

AFL, atrial flutter; GDMT, guideline-directed medical therapy; LVEDD, left ventricular end-diastolic diameter; LVEF, left ventricular ejection fraction; NT-proBNP, N-terminal prohormone of B-type natriuretic peptide.

© 2026 Li S et al. *JAMA Network Open*.

eFigure 1. Number of Patients Using GDMT at Randomization and Monthly Follow-Ups

(A) GDMT Withdrawal Group

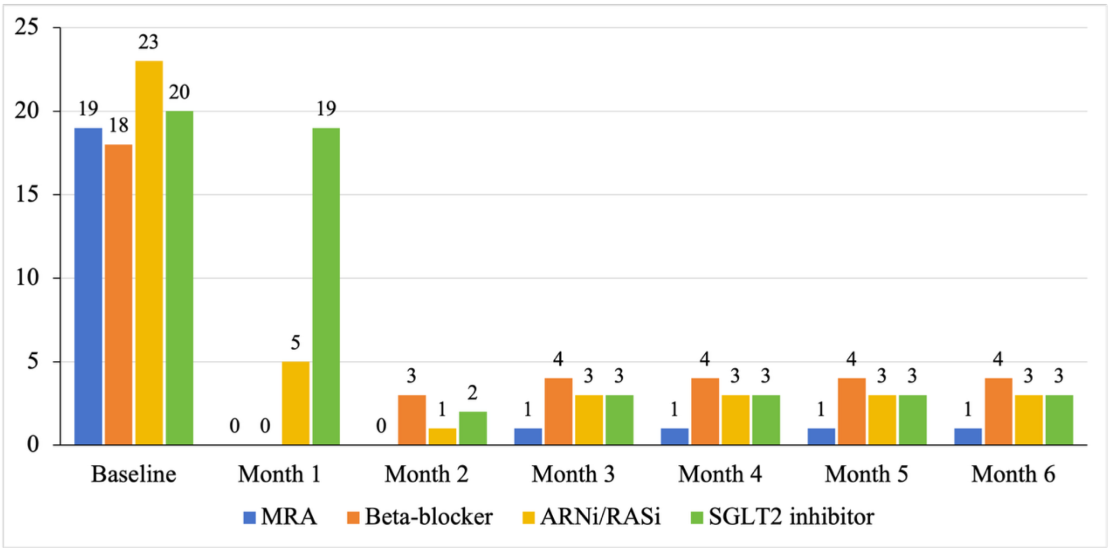

(B) GDMT Continuation Group

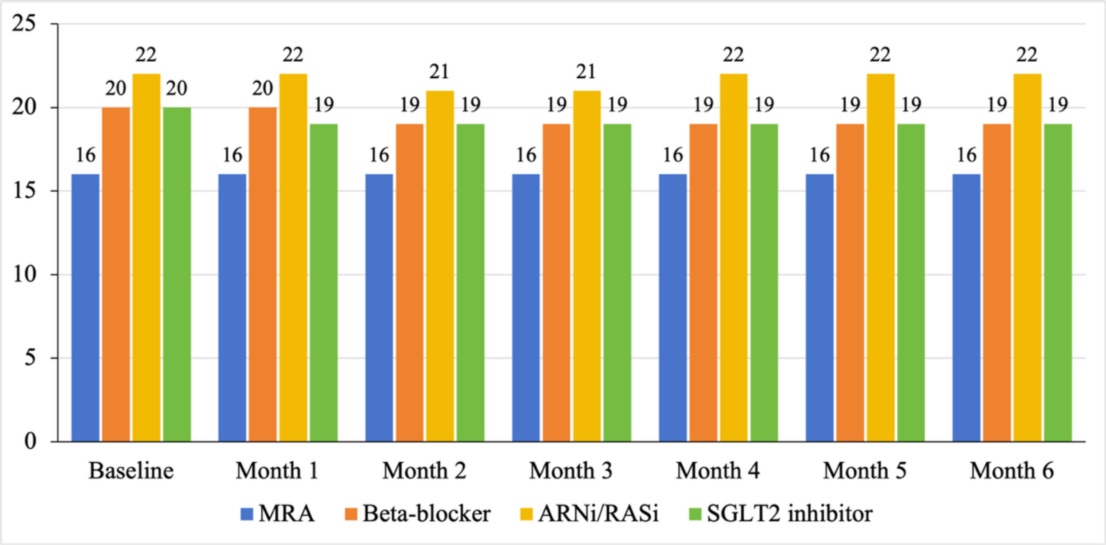

(A) indicated patients in the GDMT withdrawal group, and (B) indicated patients in the GDMT continuation group.

ARNi, angiotensin receptor-neprilysin inhibitor; MRA, mineralocorticoid receptor antagonist; RASi, Renin-angiotensin-system inhibitor; SGLT2, sodium-glucose cotransporter 2.

© 2026 Li S et al. *JAMA Network Open.*

eFigure 2. Normal Q-Q Plots for Changes in Echocardiographic LVEF, LVEDD, NT-proBNP Level, and KCCQ-12 Score by GDMT Groups

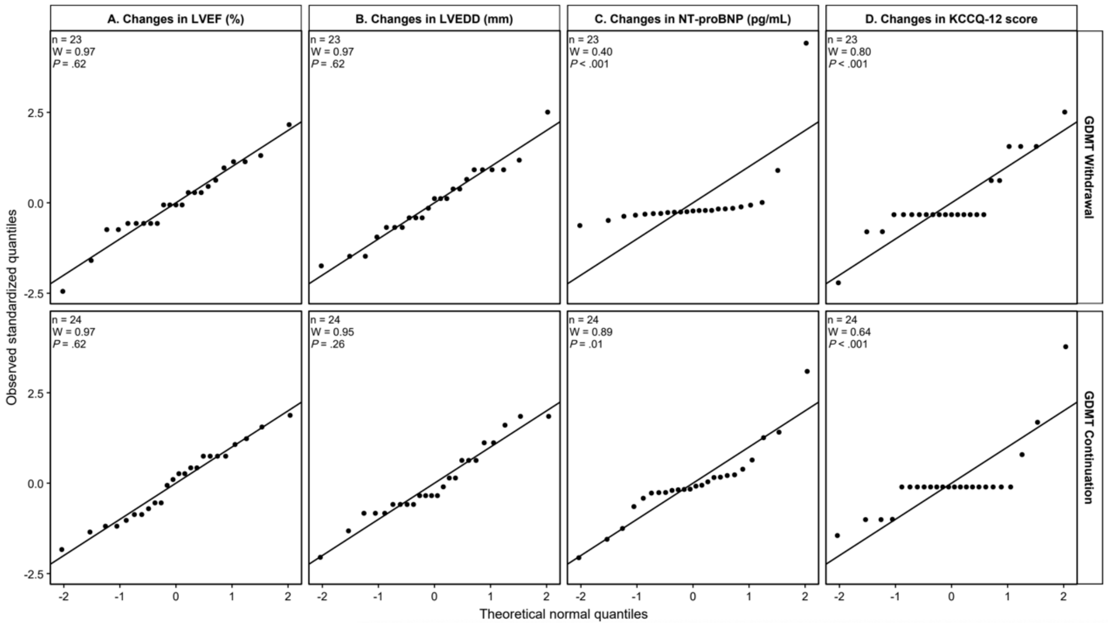

GDMT, guideline-directed medical therapy; KCCQ-12, Kansas City Cardiomyopathy Questionnaire-12; LVEDD, left ventricular end-diastolic diameter; LVEF, left ventricular ejection fraction; NT-proBNP, N-terminal prohormone of B-type natriuretic peptide.

© 2026 Li S et al. *JAMA Network Open.*

eFigure 3. Normal Q-Q Plots for Changes in LVEF, LVEDVi, LV-GLS, and LGE on CMR

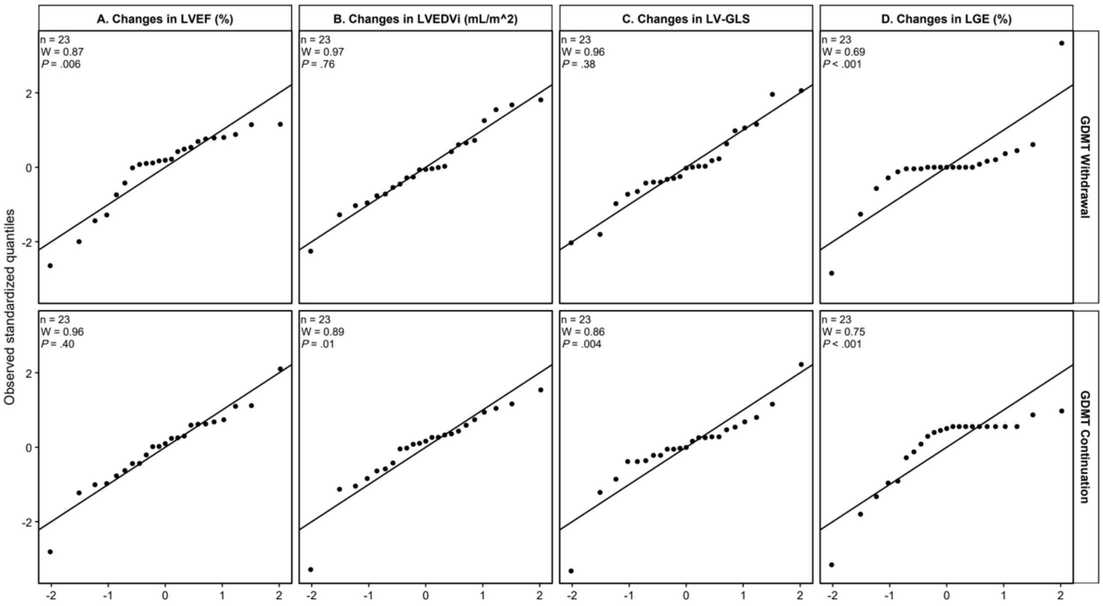

GDMT, guideline-directed medical therapy; LGE, late gadolinium enhancement; LVEDVi, left ventricular end-diastolic volume index; LVEF, left ventricular ejection fraction; LV-GLS, left ventricular global longitudinal strain.

© 2026 Li S et al. *JAMA Network Open*.
