# Supplementary material for: Heart Failure Medication Withdrawal in Patients With Improved Cardiac Function After Atrial Fibrillation Ablation: The DEFINITION-AF Pilot Randomized Clinical Trial
Source: JAMA Netw Open. 2026 Jun 26;9(6):e2620145. doi: 10.1001/jamanetworkopen.2026.20145 (PMC13309871; doi:10.1001/jamanetworkopen.2026.20145)
Supplement: Supplement 3. — Data Sharing Statement [file jamanetwopen-e2620145-s003.pdf]

Data Sharing Statement

Li. Heart Failure Medication Withdrawal in Patients With Improved Cardiac Function After Atrial Fibrillation Ablation. *JAMA Netw Open*. Published June 26, 2026.
doi:10.1001/jamanetworkopen.2026.20145

Data
Additional Information: chictr.org.cn URL: https://www.chictr.org.cn/showproj.html?proj=194776 Identifier: ChiCTR2300077439.
Data available: Yes
Data types: Deidentified participant data
How to access data: Individual de-identified participant data will be available upon reasonable request from the corresponding authors
When available: With publication

Supporting Documents
Document types: None

Additional Information
Who can access the data: Researchers whose proposed use of the data has been approved
Types of analyses: For academic purpose
Mechanisms of data availability: After approval of a proposal
